# Lithium isotope evidence for enhanced weathering and erosion during the Paleocene-Eocene Thermal Maximum

**DOI:** 10.1126/sciadv.abh4224

**Published:** 2021-10-15

**Authors:** Philip A. E. Pogge von Strandmann, Morgan T. Jones, A. Joshua West, Melissa J. Murphy, Ella W. Stokke, Gary Tarbuck, David J. Wilson, Christopher R. Pearce, Daniela N. Schmidt

**Affiliations:** 1 Institute of Geosciences, Johannes Gutenberg University, 55122 Mainz, Germany.; 2 London Geochemistry and Isotope Centre (LOGIC), Institute of Earth and Planetary Sciences, University College London and Birkbeck, University of London, Gower Place, London WC1E 6BS, UK.; 3 Centre for Earth Evolution and Dynamics (CEED), University of Oslo, Pb. 1028 Blindern, 0315 Oslo, Norway.; 4 Department of Earth Sciences, University of Southern California, 3651 Trousdale Parkway—ZHS 117, Los Angeles, CA 90089, USA.; 5 Centre for Permafrost, University of Copenhagen, Copenhagen, Denmark.; 6 National Oceanography Centre Southampton, University of Southampton Waterfront Campus, European Way, Southampton SO14 3ZH, UK.; 7 School of Earth Sciences, University of Bristol, Wills Memorial Building, Queens Road, Bristol BS8 1RJ, UK.

## Abstract

The Paleocene-Eocene Thermal Maximum (PETM; ~55.9 Ma) was a geologically rapid warming period associated with carbon release, which caused a marked increase in the hydrological cycle. Here, we use lithium (Li) isotopes to assess the global change in weathering regime, a critical carbon drawdown mechanism, across the PETM. We find a negative Li isotope excursion of ~3‰ in both global seawater (marine carbonates) and in local weathering inputs (detrital shales). This is consistent with a very large delivery of clays to the oceans or a shift in the weathering regime toward higher physical erosion rates and sediment fluxes. Our seawater records are best explained by increases in global erosion rates of ~2× to 3× over 100 ka, combined with model-derived weathering increases of 50 to 60% compared to prewarming values. Such increases in weathering and erosion would have supported enhanced carbon burial, as both carbonate and organic carbon, thereby stabilizing climate.

## Teaser

Silicate weathering and physical erosion markedly increased during the rapid warming of the PETM, allowing climatic recovery.

## INTRODUCTION

The Paleocene-Eocene Thermal Maximum (PETM) was a geologically rapid hyperthermal event that occurred around 55.9 million years (Ma) ago. It was associated with substantial release of carbon to the atmosphere, which caused up to 8°C surface ocean warming in high latitudes (*1*, *2*). The environmental changes included rapid warming, ocean acidification, and perturbations to the hydrological cycle, which resulted in a major biotic turnover in the surface ocean and mass extinction in the deep ocean (*1*–*3*). While the sequence of temperature changes across the event is well studied, the mechanisms leading to the climatic recovery have received less attention.

The ultimate cause and duration of the carbon release is still debated, but the interpretation of several sedimentary records suggests that the negative carbon isotope excursion (CIE) had a rapid “onset phase” of around 1 to 5 ka, a “body phase” of around 70 to 100 ka characterized by stable but anomalously low δ^13^C values, and a “recovery phase” of another 50 to 100 ka (*4*). The mechanisms responsible for the recovery from this carbon release and warming are poorly understood, but likely involved chemical weathering of silicate rocks and/or burial of organic carbon. Chemical weathering sequesters CO_2_ by increasing the supply of dissolved cations (such as Ca and Mg) and alkalinity to the oceans (*3*, *5*–*8*). This change in ocean chemistry, leading to marine carbonate precipitation and burial, is described as a carbonate preservation overshoot (*9*). Higher weathering inputs also increase the availability of dissolved nutrients (Fe, P, Si, etc.) that trigger organic carbon production in the coastal oceans (*10*, *11*). Although climate-forced changes in chemical weathering have been proposed as a potential feedback mechanism driving climatic recovery from the PETM (*3*), it is not clear whether the rate of weathering and consequent carbonate precipitation was rapid enough to explain the rate of recovery (*12*). Earth System models of intermediate complexity (EMICs) have suggested that chemical weathering can account for the entirety of carbon drawdown, although not the associated carbon isotope ratios (*3*).

Increased weathering (*13*–*16*) and organic carbon burial may have been driven not only by a warmer climate but also by increases in erosion and sediment accumulation rates, which are documented across the PETM (*1*, *4*, *12*). In addition to increased sedimentation rates, several localities show spikes in kaolinite abundance in sediments at the PETM, which have been attributed to increased erosion of terrestrial clays formed before the PETM, primarily by riverine incision (*1*, *17*–*19*). Increased erosion may drive higher chemical weathering fluxes (and hence CO_2_ drawdown), as documented in modern river systems (*20*, *21*). Furthermore, organic carbon burial is also greatly enhanced by the presence of clay particles, which inhibit reoxidation of the organic matter and are created on the continents through chemical weathering and delivered to the coastal oceans via physical erosion (*10*, *11*). However, the links between an intensified hydrological cycle, enhanced erosion fluxes, and CO_2_ drawdown at the PETM are not well understood. More generally, while there are widespread local records of changes in sedimentation rate, globally integrated changes in erosion or sedimentation rates across the PETM are lacking to date. New evidence is therefore needed to test the hypothesis of a major global increase in erosion rates during the PETM and its role in the carbon cycle recovery.

Here, we use Li isotope ratios to determine how silicate weathering rates and weathering regimes (i.e., the balance of chemical weathering to physical erosion) changed during this hyperthermal event. Unlike many other geochemical signatures, Li isotopes provide a global weathering proxy that is dominantly controlled by silicate weathering processes. Although evaporite weathering can affect the local composition of some surface waters, on a global scale these sources appear to have relatively little impact on the seawater Li isotope compositions (*22*). In river water, Li isotopes are fractionated according to the ratio of primary rock dissolution (which does not fractionate Li isotopes, and hence drives riverine δ^7^Li to low values) to clay formation (driving δ^7^Li to high values), which is known as the weathering congruency (*23*, *24*). This congruency can also be interpreted in terms of silicate weathering intensity *W*/*D*, which is the ratio of the chemical weathering (*W*) rate to the total [erosion (*E*) + chemical weathering (*W*)] denudation rate (*D*) (*25*). Note that, here, “erosion” represents the combination of erosion of bedrock, along with remobilization and net erosion of riverbank sediments, for example, in floodplains (*8*, *25*). This is effectively the riverine sediment flux, but given that the definition of erosion (e.g., by the Geological Society of London) includes the movement of both rock and soil (*20*, *26*), we use the term erosion throughout. Low-intensity regimes are characterized by high primary rock dissolution relative to clay formation, and hence low riverine δ^7^Li, but also high dissolved Li fluxes. Moderate intensity regimes exhibit increased clay formation (i.e., incongruent weathering), and hence high riverine δ^7^Li, but lower Li fluxes. In high-intensity regimes, there is little primary rock dissolution, and instead secondary clays dissolve, leading to a low δ^7^Li, but also a low Li flux (*8*, *25*). Rivers make up ~60% of the Li inputs to the modern oceans (where Li has a ~1-Ma residence time), with the remainder from mid-ocean ridge hydrothermal solutions (*27*), which also agrees with overall ridge water fluxes (*28*). In this study, we analyzed Li isotope ratios from multiple global carbonate sections across the PETM (both bulk planktonic foraminifera and bulk carbonates), as well as detrital shale records. By identifying common trends across sections, as well as considering trace element ratios, we minimize artifacts associated with diagenesis or other local effects that may alter Li isotope ratios (*29*).

## RESULTS

### Carbonate sections

**Fig. 1.**
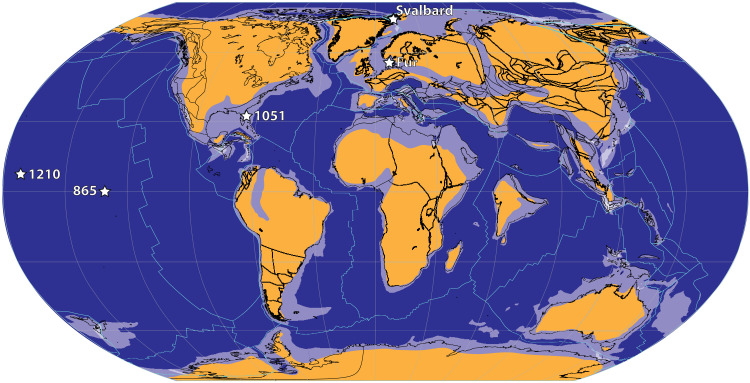
Sample location map on a paleomap from the PETM. Sites 865, 1051, and 1210 refer to Ocean Drilling Program sites, from which we analyze carbonates. Svalbard and Fur are detrital shale sample sites. See the Supplementary Materials for sample details.

We report Li isotope ratios from three marine carbonate sections [bulk carbonate: Ocean Drilling Program (ODP) sites 865 and 1051; bulk foraminifera: ODP 1210; Fig. 1; see also the Supplementary Materials] and from two detrital shale sections (Svalbard and Fur, Denmark). In all three carbonate ODP sites, Al/Ca and Rb/Ca values indicate that insignificant amounts of leaching of silicate material occurred (see the Supplementary Materials). An initial consideration is whether the cores are affected by diagenesis. A recent detailed study has examined the effects of diagenesis on carbonate Li isotopes (*29*), proposing target elemental ratios that may indicate diagenetic alteration of the Li system. Our bulk carbonate Li/(Ca + Mg) values (12 to 40 μmol/mol) generally fit within the suggested modern range for bulk carbonates of 20 to 40 μmol/mol, and our few samples with lower Li/(Ca + Mg) values are not associated with any difference in δ^7^Li values (fig. S5). Combined with Sr/Ca values of <2.6 mmol/mol, these observations suggest that our samples have not been significantly diagenetically altered. We note that there is typically a fractionation factor between bulk carbonate and seawater Δ^7^Li_carb-SW_ (δ^7^Li_carb_ − δ^7^Li_SW_) of −6.1 ± 1.3‰ (*7*), and we apply this offset to our data.

The diagenetically unaltered range of Li/(Ca + Mg) suggested for modern foraminifera is 12 to 16 μmol/mol (*29*). Our foraminifera record exhibits values mainly between 2.5 and 23 μmol/mol, but also includes some higher values at the bottom of our sampling interval for one core. However, as for the bulk carbonates, there is no trend between foraminiferal δ^7^Li and Li/(Ca + Mg) (fig. S5). The suggested Δ^7^Li_carb-SW_ range for foraminifera is −4 to −0‰ (*29*), around 1 to 2‰ offset from bulk carbonates. The possibility of Li incorporation into Mn oxyhydroxides, which could bias the measured δ^7^Li, can be discounted by the absence of any relationship between δ^7^Li and Mn/Ca in any of the cores (fig. S3).

While there is general agreement that there is no temperature effect on Li isotope fractionation in calcite (*6*, *7*, *30*, *31*), a negative relationship between pH and calcite δ^7^Li has been reported for some epibenthic foraminifera (*30*) and inorganic calcite (*32*). However, other studies directly contradict this (*31*), leaving an uncertainty in the potential influence of pH on δ^7^Li. No such effect has been documented for planktonic foraminifera or coccoliths (*7*, *24*, *27*, *29*). Different planktonic foraminifera species have been reported to exhibit offsets in Li isotopic fractionation factors (*27*), although this is also disputed (*33*), and there is no covariation between δ^7^Li_foram_ and species diversity (see the Supplementary Materials). However, even if the reported pH effect on epibenthic foraminifera did affect bulk carbonates, we would anticipate a pH-associated positive δ^7^Li excursion (LIE) of ~0.9‰ for the PETM pH decrease (*3*, *30*), which is opposite to the changes observed here, implying that the actual excursion in seawater would, if anything, have been larger than seen in our records.

**Fig. 2.**
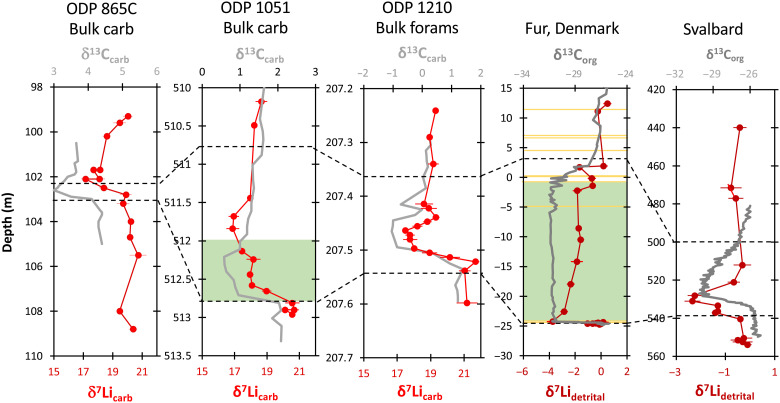
Lithium and carbon isotope records from the studied carbonate and shale sections. Sites 865, 1051, and 1210 represent marine carbonates, while the Fur and Svalbard sections are shales (analyzed in bulk for Li isotopes). The dashed lines represent the onset and termination of the PETM. For the site 1051 and Fur sections, the green band is the duration of the reported enhanced organic matter burial (*79*, *80*). For the Fur section, the yellow lines represent ash layers (*80*). The error bars on the Li isotope data represent the 2 SD precision on each analysis. External precision was ±0.4‰.

Because three different carbonate sections from different locations reproduce similar values and temporal patterns (Fig. 2), based on both bulk carbonates (~95% coccoliths in these cores, where modern core-top studies also show no difference in δ^7^Li or Li/Ca in cores with different fractions of coccoliths versus foraminifera; fig. S6) (*7*) and separated foraminifera, we are confident that these records are reliably recording paleo-seawater δ^7^Li values. When compared to the two ODP bulk carbonate sections, the slightly higher absolute δ^7^Li values recorded by the foraminifera at site 1210 are consistent with a slightly lower isotopic fractionation factor in foraminifera compared to bulk carbonate (*29*). Therefore, identical seawater values are reconstructed from all sites, with δ^7^Li_seawater_ ~ 26.4 to 26.8‰ before the PETM, which agrees with the fractionation-corrected Cenozoic foraminiferal record (*24*). All sections indicate a ~3 to 4‰ negative LIE in seawater during the PETM (Fig. 2). While the long-term Cenozoic Li isotope record (*24*) is sparse near the PETM (average resolution of one data point every 1.5 Ma), it does include ~60-ka resolution between 59.9 and 62 Ma, with a variability of ±1.2‰. Notably, the PETM δ^7^Li perturbation of 3 to 4‰ stands out from relatively uniform data during the preceding several million years.

### Shale sections

Shale sections, especially those with a high detrital content such as those analyzed here, should record more local Li isotope compositions, i.e., the signal originating from weathering of the continents (*34*, *35*). The Fur shale section was deposited in an epicontinental sea, with heavily restricted access to the open ocean (Fig. 1). The Svalbard section was deposited in more open water, but this area of the Arctic was still limited to exchange with other oceans at this time. Detrital clays are associated with weathering processes at the scale of individual river basins that delivered the detrital material to continental shelves. Absolute δ^7^Li values are subject to a secondary mineral fractionation factor, with a global average Δ^7^Li of ~15‰, although precise mineralogy-specific fractionation factors are not yet well known (*27*, *36*, *37*). Detrital clays have been used as a local Li archive in several different paleo-settings (*34*, *35*, *38*), although the caveat is that the local Li signal recorded in clay minerals is likely to be diluted by previously formed clay minerals eroded from the continents. This means that any signal recorded in shales is likely to represent a minimum excursion. Nonetheless, the shale sections studied here also show a negative 2 to 3.5‰ LIE, similar to that seen in the carbonates in this study. In all shale and carbonate sections, the LIEs appear to start at the onset of the CIE (Fig. 2), and at Fur also contemporaneously with the PETM sea-surface temperature increase (fig. S16) (*39*).

The δ^7^Li variability in shale sections could represent a change in river basin–scale water composition due to changes in weathering congruency or an indirect effect of a changing weathering regime or primary lithology via changing clay mineralogy (*8*, *19*, *34*, *35*, *37*, *38*). In both sections, there is a PETM peak in kaolinite abundance that is interpreted to indicate enhanced erosion rates, transporting previously formed clays from the continents to the oceans (fig. S16) (*14*, *17*–*19*). However, there is no statistically significant correlation (*P* > 0.05, Spearman rank) between δ^7^Li and clay abundances (fig. S15) or between δ^7^Li and the chemical index of alteration (fig. S16) (*19*, *40*). Therefore, the LIE was not simply caused by a change in clay mineralogy. Detrital osmium isotope ratios in the Svalbard section (*40*) also exhibit a different temporal pattern from the Li isotopes (fig. S17), which argues against a change in provenance as a cause of the δ^7^Li trend. These observations therefore suggest that the decrease in shale δ^7^Li at least partly represents a decrease in river catchment–scale water δ^7^Li compositions, indicating more congruent weathering.

### Excursion timing

Our longest duration record is from site 865, which extends both ~450 ka before and ~600 ka after the CIE initiation (*41*). In the carbonate sections, the LIE appears to last considerably longer than the CIE, which reflects the long ocean residence time of Li. Two of the carbonate sections do not span a sufficient amount of time to show an LIE recovery. In contrast, at site 865, pre-excursion δ^7^Li values are reached again in the final two measured samples, ~500 to 600 ka after the PETM initiation. This time scale is slightly shorter than the modern oceanic Li residence time for Li of ~1 Ma. We note, however, that the Li residence time in seawater may have been shorter in the past, as suggested for both Li and Sr during Oceanic Anoxic Event 2 at ~94 Ma (*42*), and discussed further below.

In contrast, in the shale sections, the LIE is shorter than the CIE, which provides a better indication for the duration of the weathering change, because Li isotope changes in detrital material are not buffered by the long oceanic Li residence time. Floodplain sediment transit could buffer the transmission of detrital weathering signals to sedimentary depocenters, although even for large floodplains in the present day, the transit time for fine-grained material is thought to be ~10 years to <100 ka (*43*), so a detrital LIE response that is shorter than the 120- to 200-ka CIE is not surprising.

## DISCUSSION

Our carbonate records suggest a pre-PETM seawater δ^7^Li value of ~26.6 ± 1.3‰ [where the uncertainty stems from uncertainty in the fractionation factor into carbonate (*7*)], in agreement with the long-term fractionation-corrected Cenozoic Li isotope record (*24*, *29*), and a peak LIE δ^7^Li value of 23 to 24‰ during the PETM. While there is no known temperature effect on Li isotope fractionation during bulk calcite mineralization (*6*, *7*, *30*–*32*), there is a temperature effect on the fractionation factor imposed during Li uptake into secondary minerals (*36*). This fractionation would affect river water δ^7^Li (i.e., higher temperatures will cause river water δ^7^Li to be lower), as well as seawater δ^7^Li (i.e., during reverse weathering, higher temperatures will cause lower seawater δ^7^Li), independently from any change in chemical weathering. Combined, fractionation during weathering and reverse weathering give a temperature dependence of −0.25‰/K for seawater Li (*36*). Thus, an overall 4° to 8°C continental warming (*2*) during the PETM would cause rivers to become 0.6 to 1.4‰ (average, ~0.9‰) lighter and decrease the δ^7^Li of the overall ocean input by 0.5 to 1.2‰ (average, ~0.8‰). Bottom ocean warming of 4° to 5°C (*2*, *44*) would result in a muted fractionation between seawater and the clay Li sink, but this effect would be buffered by the ocean residence time. Overall, the warming would be expected to cause a δ^7^Li decrease of ~1 to 1.5‰ instantaneously in marine carbonates (due to the riverine source composition) and 1.5 to 2‰ in marine carbonates overall. In detrital shales, the temperature effects from continental weathering and from the authigenic formation of secondary clays would approximately balance out. Therefore, the excursion exhibited by the shales is interpreted as a 2 to 3.5‰ decrease in δ^7^Li of the local catchment-scale solutions around Denmark and Svalbard. In contrast, of the ~3‰ LIE recorded in the marine carbonates, only ~1 to 1.5‰ likely represent an actual change in seawater δ^7^Li due to a shift in weathering, while the rest is interpreted to be a temperature-related effect. Together, we suggest that changes in silicate weathering are responsible for a shift in seawater δ^7^Li compositions from ~26‰ before the onset of the PETM to 24.5 to 25.0‰ during the PETM.

### Modeling the Li isotope excursion

We model the seawater LIE using dynamic box models that have been used with varying complexity for modeling other time periods (*34*, *42*, *45*). As a starting point for modeling the seawater Li isotope evolution, we assume that the hydrothermal input can be constrained from mid-ocean ridge spreading rates, with the PETM hydrothermal input between 1.15× and 1.4× that of the present (*46*, *47*). The maximum effect of this range in hydrothermal input on steady-state seawater δ^7^Li is 0.5‰. For hydrothermal input to be the cause of the excursion would require a transient >2× to 3× increase in hydrothermal input (see the Supplementary Materials), which is unfeasible (*40*, *48*). For the remaining models, we use an intermediate hydrothermal input of 1.25× present.

**Fig. 3.**
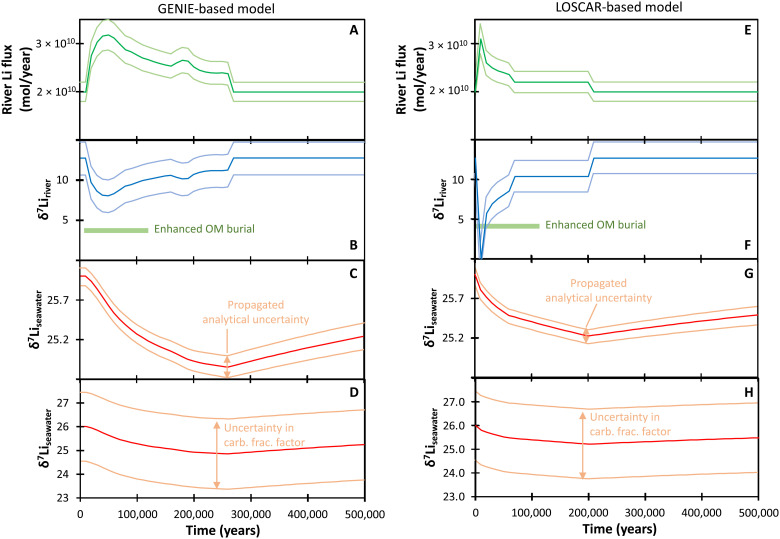
Model inputs (river Li flux) and outputs (riverine δ^7^Li) required to cause a ~1‰ excursion in seawater. Different seawater δ^7^Li uncertainty bands are shown (**C**, **D**, **G**, **H**), based both on the uncertainty in the perturbed riverine δ^7^Li (**B**, **F**; effectively the analytical uncertainty on our measured data), and on the ±1.5‰ uncertainty in the carbonate fractionation factor in foraminifera (*29*). The uncertainty bands in the riverine δ^7^Li (B, F) show the uncertainty necessary to account for the variation in the carbonate fractionation factor. This is a maximum estimate in the uncertainty, as foraminifera assemblages were more constant in our samples (see the Supplementary Materials). The green bars in panels B and F indicate the period of elevated organic matter (OM) burial at site 1051 (*79*).

To model the isotope excursion, we use the silicate weathering outputs from two Earth System models that were previously run for the PETM: a GENIE EMIC model (*3*) and a LOSCAR box model (*5*) (Fig. 3 and the Supplementary Materials). Both provide a time-resolved output of seawater chemistry changes (which is what the Li isotope data are recording), as well as marine carbon isotope and *P*co_2_ (partial pressure of CO_2_) values. The models differ in how they parameterize the silicate weathering response to climate warming. In cGENIE, weathering is controlled by the RoKGeM model, where the weathering rate is a function of both temperature and water-rock interaction time (reflected in continental runoff). In LOSCAR, the silicate weathering rate is parameterized directly as a function of *P*co_2_. The strength of the weathering feedback and the initial (steady-state) weathering flux are also controlled by the model (*49*, *50*). This allowed initial testing of the feedback strength for the PETM in the original model (*5*, *9*), effectively allowing parameterization of regolith thickness. Both models suggest an initial (steady-state) weathering flux similar to or slightly higher than the present day, which also agrees with modeled Cenozoic weathering patterns (*36*). Some interpretations of radiogenic isotope ratios in seawater have suggested that the global weathering flux was low before the PETM due to highly peneplained continents (*47*). Such a scenario is also tested here (see the Supplementary Materials), but it results in an insufficient control of riverine δ^7^Li on seawater δ^7^Li to be able to generate a seawater excursion of the observed magnitude.

The cGENIE model calculates a 60% increase in silicate weathering across the PETM, peaking at 50 ka after initiation (*3*). The LOSCAR model suggests a 55% increase, but peaking over a shorter time period of 10 ka (Fig. 3) (*5*). Both results are similar to estimates of increased weathering from both marine osmium and silicon isotope records (*48*, *51*). Changes of this magnitude have been documented in both local and global modern rivers for a 3° to 4°C temperature increase (*20*, *21*, *52*). An increase in chemical weathering at the PETM is also consistent with increased marine carbonate burial during the recovery period following the PETM (*53*).

**Fig. 4.**
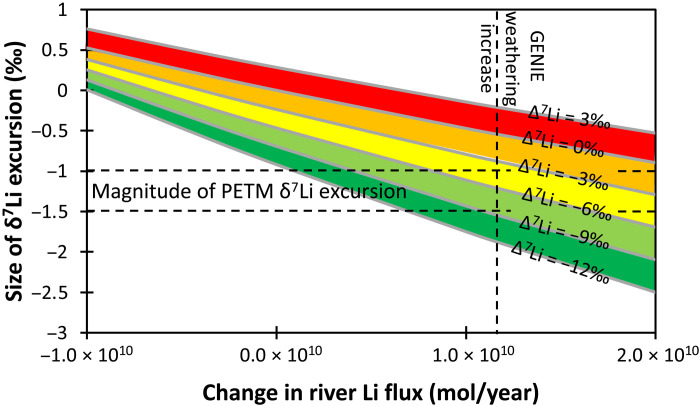
Contour plot showing the change in riverine Li flux (*x* axis) and isotope ratio (contours) required to create a seawater Li isotope excursion of a given magnitude (*y* axis). The basic model parameters for this sensitivity study are those of our GENIE-based model (see text for details). The contours represent Δ^7^Li_during-pre_ (i.e., the change from before to during the PETM in rivers) such that a contour value of −12‰ is a decrease from 13‰ pre-PETM to 1‰ during the PETM, while +3‰ is an increase from 13‰ pre-PETM to 16‰ during the PETM. The horizontal dashed lines represent the observed magnitude of the Li isotope excursion that is attributable to weathering, while the vertical dashed line represents the weathering increase suggested by the GENIE model (*3*). The observed negative seawater LIE at the PETM can only be caused by both an increase in river flux and a decrease in river δ^7^Li.

**Fig. 5.**
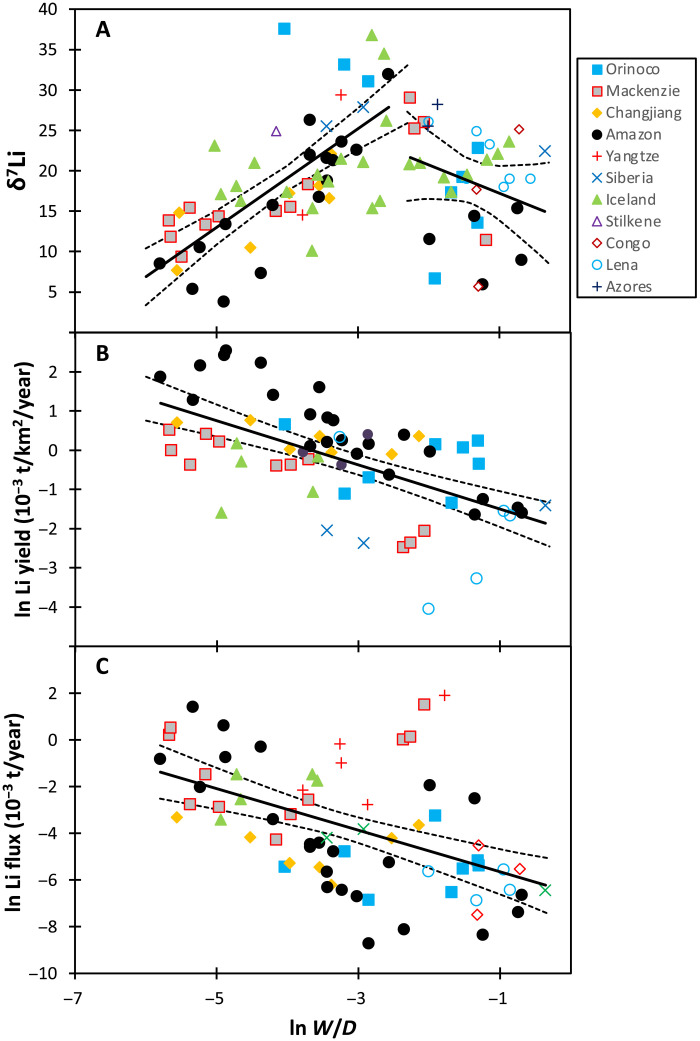
Modern riverine Li isotope data. (See section S6 for references). (**A**) Shows riverine- δ^7^Li as a function of the natural logarithm of the weathering intensity (W/D). (**B**) Shows the natural logarithm of the weathering intensity versus the log of the Li yield and (**C**) shows the natural logarithm of the riverine Li flux as a function of the log of the weathering intensity. The solid lines are the regression lines, and the dashed lines represent the 95% confidence limits, which are also used to estimate uncertainty in Fig. 6. In (A), trends are shown separately for low- to medium-intensity and high-intensity weathering regimes (*25*). Combined, this figure suggests that an increase in Li flux (and Li yield) and a decrease in riverine δ^7^Li all imply lower weathering intensity (i.e., relatively higher erosion rates) (*25*).

Assuming that the global mean riverine δ^7^Li composition stayed constant during the PETM, a global weathering rate increase of ~50% would cause a 0.4‰ transient decrease in seawater δ^7^Li (Fig. 4), considerably smaller than the observed changes in our carbonate records. Driving a transient ~1‰ decrease in seawater δ^7^Li (i.e., the minimum observed isotopic shift after accounting for the temperature-dependent isotope fractionation) requires an increase in the riverine Li flux of 55 to 60%, combined with a transient decrease in riverine δ^7^Li (Fig. 4). This behavior has also been invoked for oceanic anoxic events (*42*, *45*) and may be a common feature of past warming intervals. Conceptually, a coupling between the rate of increase in the riverine Li flux and the rate of decrease in the Li isotope ratio makes sense given that clay formation will both decrease the riverine Li flux and increase its δ^7^Li, as observed in modern rivers (Fig. 5) (*25*). The cGENIE-based model can achieve the observed decrease in δ^7^Li_SW_ with a ~5‰ decrease in riverine δ^7^Li, reaching its minimum after 50 ka (Fig. 3). In contrast, the LOSCAR-based model achieves its weathering maximum after 10 ka, but because of this shorter duration, requires a brief 12‰ decrease in river δ^7^Li (Fig. 3). This decrease would involve riverine δ^7^Li reaching the likely minimum possible value of rivers weathering primary silicates, equaling that of continental crust [~0‰ (*54*)], and yet still cannot cause more than a 0.8‰ seawater excursion. Uncertainty in the model’s seawater δ^7^Li values is determined by analytical uncertainty in the measured carbonate δ^7^Li values (Fig. 3, C and G) and separately by the carbonate fractionation factor (Fig. 3, D and H) (*7*, *29*). The latter larger uncertainty has been propagated backward to the riverine flux and δ^7^Li values (Fig. 3, A, B, E, and F).

In all cases, the initial downward limb of the PETM LIE is resolvable in both models and data, despite the long ocean residence time of Li, because the excursion is caused by Li inputs that increase the seawater Li concentration. The major effect of seawater buffering is to slow the Li isotope recovery after the perturbation (see fig. S9) (*34*, *42*). Using a modern ocean residence time, seawater δ^7^Li would recover to within ~0.4 to 0.5‰ of pre-excursion values after ~600 ka (i.e., the apparent length of the LIE at site 865). This slight offset from pre-excursion values is within the analytical uncertainty on the carbonate data, and therefore, the data and model outputs are consistent (see fig. S9 for effects of changing residence times).

### Changes in weathering regime across the PETM

The model results show that changes in weathering flux alone cannot explain the observed excursions in seawater δ^7^Li across the PETM, and substantial changes in the isotope ratio of the inputs to the oceans are also required. There are several obvious possible mechanisms for explaining such changes. One possibility is that erosion of Cretaceous laterites, which are thought to have been the source of abundant kaolinite in PETM sedimentary records (*1*), supplied a large amount of isotopically light clays to floodplains and the oceans. Dissolution of these clays would contribute isotopically light Li (i.e., the riverine δ^7^Li becomes similar to the values observed in our detrital LIE), potentially driving the negative excursion. Unlike in modern rivers, where clay dissolution is accompanied by low Li fluxes (Fig. 5), in this case, Li fluxes from clay dissolution would have to have been high. Critically, for the clays to have affected seawater δ^7^Li during the PETM, they would have needed to dissolve or otherwise react in seawater to release their isotopically light Li. To cause the observed isotope excursion through dissolution of isotopically light clay, the cGENIE-derived model requires that dissolution increases by >1.1× (fig. S12). If this material was laterite as suggested [5 to 10 μg/g Li (*55*)], then clay dissolution would need to have reached values of ~8 to 15 Gt/year. If, for the sake of argument, the source material were saprolite [~15 to 50 μg/g Li (*56*, *57*)], then ~2 to 6 Gt/year of clay would need to dissolve in the oceans. For comparison, the pre-anthropogenic sediment flux (i.e., before dissolution) to the oceans is estimated at ~20 Gt/year (*26*). Normalized to surface area, clays such as kaolinite and smectite have dissolution rates that are one to three orders of magnitude lower than primary minerals such as olivine or plagioclase [e.g., albite: ~10^−11^ mol g^−1^ s^−1^ (*58*, *59*); olivine: ~10^−10^ mol g^−1^ s^−1^ (*60*); kaolinite: ~10^−12^ to 10^−13^ mol g^−1^ s^−1^ (*61*–*63*); montmorillonite: ~10^−12^ mol g^−1^ s^−1^ (*61*, *64*)]. This would then suggest that the PETM clay supply to the oceans was considerably higher (potentially by an order of magnitude or more) than at the present day. While this is possible and cannot be discounted, given the sedimentation rate increases discussed below, we suggest that this would require a larger increase in the global erosion rate (river sediment flux) than the alternatives presented below.

An alternative explanation for the change in riverine δ^7^Li at the PETM, and one we consider more likely, is that weathering intensity and fluxes changed in response to the climatic perturbation during this event. In present-day rivers, weathering intensity, Li fluxes, and Li isotope ratios are related (Fig. 5) (*8*, *25*), and mass balance suggests that similar relationships must have existed in the Cretaceous (*42*) and Hirnantian (*34*). Following the relationship seen in these rivers, coupled changes in riverine Li flux and δ^7^Li could result from an adjustment in the silicate weathering regime and intensity, i.e., the ratio of weathering rate to denudation rate (*W*/*D*), where denudation is the sum of the weathering and erosion rates. Presently, low riverine δ^7^Li occurs either when *W*/*D* is very low [i.e., high erosion (sediment fluxes) relative to weathering rates] or when *W*/*D* is very high (i.e., supply-limited chemical weathering, where previously formed clays are redissolving) (Fig. 5). The latter situation is similar to the first scenario discussed above, in which clay dissolution was considered as a potential driver for the low riverine δ^7^Li at the PETM. However, if modern river trends are followed, the riverine Li flux and yield in a high *W*/*D* world would be up to two orders of magnitude lower than in a low *W*/*D* world (Fig. 5). Such a low Li flux to the oceans would lead to dominance of the hydrothermal input (considering the modern seawater mass balance, hydrothermal fluxes would then make up ~90% of the total inputs). In this case, seawater δ^7^Li would be difficult to perturb via weathering (*23*, *25*, *65*), and the observed excursion could not be replicated (Fig. 4 and the Supplementary Materials). We therefore consider a high *W*/*D* scenario (low δ^7^Li–low flux) to be unlikely.

The more plausible explanation for the observed seawater LIE is a decrease in silicate weathering intensity to relatively low *W*/*D* values, whereby rock dissolution outpaces clay formation and dissolved δ^7^Li decreases as a result (*25*). Because weathering fluxes increased at the PETM [based on evidence from increases in clay in coastal sections (*1*), seawater Os isotope ratios (*48*), and Earth System models (*3*, *5*)], a decrease in *W*/*D* would have required an even greater increase in erosion (sediment flux) rates than the weathering increase. This agrees with the modern global large river behavior, where increasing runoff causes erosion (sediment flux) rates to increase faster than silicate weathering rates (fig. S18) (*20*). Large increases in erosion rates are consistent with major local sedimentation rate increases observed in many global locations (*1*, *14*, *66*, *67*), by over an order of magnitude in some cases, primarily due to fluvial incision as runoff increased. While these increases are heterogeneous and cannot, on their own, be used to estimate global changes in erosion rates (*68*), local increases from pre-PETM rates are as high as ~19× increase [in California (*69*)] and are frequently on the order of a 4 to 8× increase [e.g., in Uzbekistan, Kazakhstan, Spain, Tunisia, and New Jersey; see summaries in (*67*, *69*)].

**Fig. 6.**
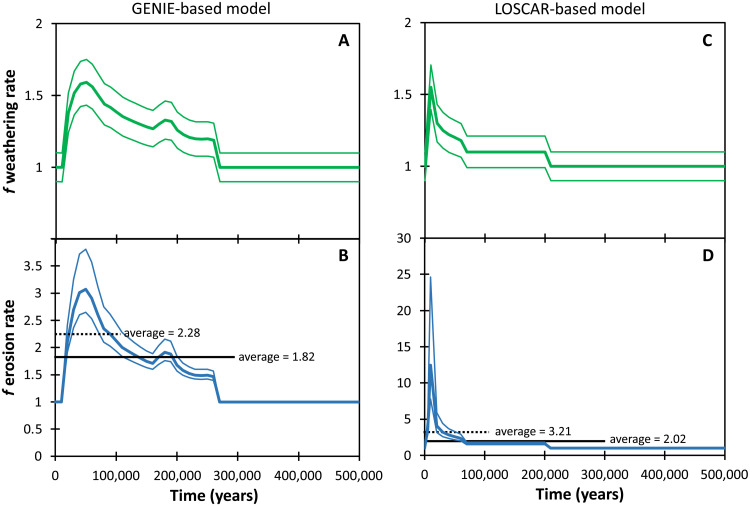
Increase in silicate weathering rate, relative to pre-PETM values. Weathering rate estimates from the GENIE (**A**) and LOSCAR (**C**) models; the corresponding increases in erosion rates are shown in (**B**) and (**D**), respectively, based on those models and the modeled and modern riverine Li isotope ratios. The uncertainty in the erosion rate stems from the uncertainty in weathering rates in the model and the uncertainty in the modern riverine δ^7^Li versus *W*/*D* curve (Fig. 5). The dotted and thick black lines represent the averages for 100- and 300-ka intervals.

On the basis of the best-fit trend from the modern global river data (Fig. 5 and the Supplementary Materials), we can estimate the increase in global erosion rates from pre-PETM to peak PETM needed to drive the inferred change in riverine δ^7^Li. On this basis, we calculate a 3.1${}_{-0.42}^{+0.80}$ times increase in erosion for the cGENIE model and a 12.5${}_{-4.7}^{+12.2}$ times increase for the LOSCAR model (Fig. 6). The uncertainty arises principally from the scatter in the modern river data (Fig. 5). For the LOSCAR model, the high erosion rate only persists for a very short period of time (<10,000 years), whereas during most of the CIE the erosion rates are slightly lower than in the cGENIE model. When averaged over 100 ka, the models generate increases in global erosion rate (i.e., the global riverine sediment flux) of 3.2× and 2.3×, respectively. The perturbation to the weathering regime lasts 200 to 300 ka, but this is largely determined by the Earth System models (*3*, *5*), because the seawater Li reservoir buffers the signal recovery.

Overall, such large increases in erosion rates (which, per definition, includes the river sediment flux) appear surprising and beyond those likely to be forced by plausible changes in the mean annual precipitation. However, climate models for the PETM, based on our modern understanding of the link between warming and extreme precipitation (*70*), suggest marked changes in extreme precipitation events (*67*, *71*, *72*), which do strongly affect erosion. In modern systems, modeling studies suggest that erosion rates increase by 1.7 to 9.9% for every 1% increase in total rainfall, if rainfall amount and intensity change together (*73*, *74*). Observational studies report that modern local erosion rates increase by up to 15% for every 1% increase in winter rainfall (*75*). Geomorphic evidence from relatively early in the PETM suggests that water discharge increased by 1.4 to 14× in Spain and that precipitation increased by at least 1.93× (*71*). Climate models point to similar increases in other areas (*76*), with some models suggesting that the incidence of extreme precipitation increased by up to 70% during the PETM onset (*67*). Scaled to modern responses of erosion rate (sediment flux) to rainfall, these hydrological changes at the PETM could have increased erosion rates by up to 16× in Spain, in response to an approximate doubling of precipitation. In this region, PETM sedimentation rates were considerably lower than in other locations, implying that erosion rates may even have increased more elsewhere (*1*). These are local effects, and it is unlikely that globally averaged erosion rates increased by this magnitude, but they provide independent evidence for large changes in erosion and sedimentation. These records therefore suggest that at least the lower bounds of our calculated increases in erosion based on the Li isotope record are plausible for such an extreme climate perturbation as the PETM.

Sustaining increases in erosion rates over very long (Ma) time scales would require changes in the geodynamic forces controlling uplift, because over these long time scales the erosional system tends toward a steady state between uplift and erosion rates. However, the PETM reflects a clear disequilibrium state, in both climate and related surface processes such a fluvial incision. Our model results based on the Li isotope excursion suggest that erosional disequilibrium due to fluvial incision, whether of primary material or clays, persisted for several hundreds of thousand years. Independent models of the non–steady-state responses of erosion (riverine sediment flux) rates to changes in rainfall suggest that erosion perturbations can persist on time scales of 10^5^ to 10^6^ years (*77*), implying that the transient response of erosion rates to hydrological changes at the PETM could persist for long enough to explain the seawater Li isotope record.

In our Li isotope model results, weathering and erosion rates peak at between 10 and 50 ka after initiation of the perturbation. These time scales are longer than the estimated 1- to 5-ka (LOSCAR) and ~10-ka (cGENIE) time scale of peak CO_2_ release, reflecting the relatively long time scale over which weathering regulates the carbon cycle and *P*co_2_ levels. In comparison, the climate perturbation at the Cenomanian-Turonian boundary (Ocean Anoxic Event 2) required a silicate weathering increase of ~1.5 to 2.5× for ~300 to 500 ka combined with a decrease in riverine δ^7^Li of ~12‰ (*42*). Hence, it is perhaps not surprising that the comparable warming of the PETM could lead to a decrease in riverine δ^7^Li of a similar magnitude.

In modern rivers, chemical weathering and physical erosion rates covary (*20*, *52*, *78*), and therefore, an increase in weathering would be expected to be accompanied by an increase in both nutrient input and eroded sediment supply to the oceans. Increased weathering and erosion during the PETM would increase CO_2_ sequestration by marine carbonate precipitation and burial (*3*, *53*), but would also deliver more dissolved nutrients to the coastal oceans, potentially enhancing the production of organic matter. The productivity changes during the PETM were different in coastal systems compared to the open ocean. Coastal regions experienced an increase in productivity, while records from the open ocean reflect increased oligotrophy due to enhanced surface water stratification in response to warming. At site 1051, the peaks of local organic carbon burial [as determined from barium accumulation rates (*79*)] and modeled global erosion rates coincide (fig. S19). We also note that changes in Si isotope ratios from the same site can also be partially explained by a similar increase in weathering rates as modeled here, combined with a decrease in riverine δ^30^Si (*51*), and exhibit a minimum close to the time of maximum erosion and weathering predicted by our model. The Si isotope data also require an increase in organic carbon burial. This observation supports the idea that clay particles and reactive iron delivered to the coastal oceans by erosion may have played a critical role in enhancing organic carbon burial rates, further sequestering CO_2_ and promoting climatic recovery (*10*, *11*).

Overall, therefore, the lithium isotope data suggest that the PETM warming caused a rapid acceleration of the hydrological cycle (including extreme precipitation events), which caused both an increase in silicate weathering and a marked increase in global sediment erosion rates. This, in turn, promoted not only CO_2_ drawdown via carbonate burial but also organic carbon formation and burial, thus shifting the carbon sequestration pathway, as well as enhancing carbon sequestration rates.

## METHODS

The carbonates were analyzed for Li isotope ratios (δ^7^Li) using several different methods. At sites 865 and 1051, bulk carbonates were analyzed using a weak leach of 1 M Na acetate buffered to pH 5 with acetic acid (*34*, *42*). For site 1210, the sediment was sieved, bulk foraminifera were picked out, and two leaching methods were tested: One was identical to the leaching method described above for bulk carbonates, while the other was a traditional foraminifera cleaning method. The latter involved cracking the tests under glass slides and repeatedly ultrasonicating with deionized water and methanol to remove clay particles. These two cleaning methods yield the same Li isotope results (table S1).

In all cases, the leachates were analyzed by quadrupole inductively coupled plasma mass spectrometry (ICP-MS) to determine elemental ratios such as not only Li/Ca, Mg/Ca, and Sr/Ca but also Al/Ca and Mn/Ca, to determine whether any silicate particles had been leached. The methods are described in detail elsewhere (*7*). Briefly, samples were matrix-matched to Ca (10 μg/ml) and calibrated against a set of synthetic multielement standards (which were also doped with Na to match the addition of Na acetate). Reference material JLs-1 was run as an unknown standard to assess accuracy and precision, which was within ±7% for all concentrations. If sample Al/Ca or Mn/Ca ratios appeared too high, samples were releached and Li isotopes were reanalyzed. The use of weaker leaches systematically reduced Al and Mn concentrations, suggesting that care must be taken when leaching carbonates for Li isotopes (*7*). The shale samples were dissolved by a standard method of concentrated HF-HNO_3_-HClO_4_, followed by steps of concentrated HNO_3_ and 6 M HCl.

Samples were purified for Li with a two-column method, using AG 50W-X12 resin and dilute HCl as an eluent (*34*, *42*). Purified samples from site 865C were analyzed at Oxford University, using a Nu Instruments HR-MC-ICP-MS (high-resolution multi collector ICP-MS) and normalizing to the NIST 8545 LSVEC standard. The long-term precision of this instrument is ±0.6‰ (2 SD) (*42*). All other samples were analyzed using a Nu Plasma 3 MC-ICP-MS at the LOGIC group at University College London by normalizing to IRMM-016 bracketing standards. All samples were renormalized to LSVEC, and the analytical uncertainty was propagated to account for this. Using Nu Plasma 3 and an Aridus II desolvator, a signal intensity of ~130 pA on ^7^Li for a solution of 5 ng/ml was achieved (using 10^11^-ohm resistors and an uptake rate of ~100 μl/min). Therefore, for a solution of 0.5 ng/ml, signal intensity was still 13 pA, making it easily possible to analyze samples at this low concentration. The results of several different rock standards analyzed by this method have been reported previously (*7*), and seawater gives δ^7^Li = 31.2 ± 0.4‰ (2 SD; *n* = 19). Of particular relevance for low-concentration carbonate samples is that seawater analyzed at concentrations of 0.5 ng/ml (*n* = 6) yields a long-term analytical uncertainty of ±0.4‰ (2 SD).

## Supplementary Materials

### This PDF file includes:

Sections S1 to S10 Figs. S1 to S19 Tables S1 and S2 References
