# Supplementary material for: Lithium isotope evidence for enhanced weathering and erosion during the Paleocene-Eocene Thermal Maximum
Source: Sci Adv. 2021 Oct 15;7(42):eabh4224. doi: 10.1126/sciadv.abh4224 (PMC8519576; doi:10.1126/sciadv.abh4224)
Supplement: Supplementary file 1 — Sections S1 to S10 Figs. S1 to S19 Tables S1 and S2 References [file sciadv.abh4224_sm.pdf]

Science Advances
AAAS

Supplementary Materials for

Lithium isotope evidence for enhanced weathering and erosion during the Paleocene-Eocene Thermal Maximum

Philip A. E. Pogge von Strandmann*, Morgan T. Jones, A. Joshua West, Melissa J. Murphy, Ella W. Stokke, Gary Tarbuck, David J. Wilson, Christopher R. Pearce, Daniela N. Schmidt

*Corresponding author. Email: ppoggevo@uni-mainz.de

Published 15 October 2021, *Sci. Adv.* **7**, eabh4224 (2021)
DOI: 10.1126/sciadv.abh4224

This PDF file includes:

Sections S1 to S10
Figs. S1 to S19
Tables S1 and S2
References

1. Samples

Here we analysed both marine carbonates and detrital shales from multiple localities covering different ocean basins to determine the lithium (Li) isotope response to the PETM. Three marine carbonate sections were analysed to reconstruct seawater Li isotopes: ODP 865 (Allison Guyot, western Pacific)(*81-83*), ODP 1051 (Blake Nose, western North Atlantic)(*79, 84*), and ODP 1210 (Shatsky Rise, north-west Pacific)(*85-87*). Further, two detrital shale sections were analysed to determine Li isotope compositions associated with weathering processes at the scale of individual river basins that delivered detrital material to continental shelves.

One shale section is from the borehole Store Norske Spitsbergen Kulkompani (SNSK) BH9/05, located on the eastern flank of the Paleogene Central Basin of Spitsbergen, Svalbard (*17, 40, 88*). The Central Basin began to form as a foreland basin adjacent to the West Spitsbergen fold-and-thrust belt from around 61.8 Ma (*89*). The subsidence marks the beginning of sustained compression between Greenland and Svalbard that resulted in continuous deposition into the late Eocene and is associated with the Eurekan deformation (*90, 91*). During the Paleocene-Eocene transition, the study area was a marine shelf facies dominated by laminated shales (*92*). The Svalbard section is anomalous for PETM sections as high deposition rates precede and post-date the PETM, although deposition during the PETM CIE still shows elevated sedimentation rates (*93*).

The other shale section is from Fur Island, Denmark. In the Paleogene this locality was part of the Norwegian-Danish Basin, a marginal extension of the epicontinental North Sea Basin (*94*). The emplacement of the North Atlantic Igneous Province (NAIP) had a direct effect on local sedimentation due to the associated thermal and tectonic uplift (*95, 96*),

leading to a marine regression and increased bottom-water anoxia linked to increased isolation of the North Sea Basin. The local manifestation of the PETM interval is the Stolleklint Clay (*97*), a laminated mudstone largely devoid of benthic fauna (*98*). The onset of the PETM coincides with deposition of the Stolleklint Clay, which overlies the heavily bioturbated and glauconite-rich Holmehus Clay. While quantitative estimates of pre-PETM sedimentation rates are lacking, sedimentary features are indicative of low sedimentation rates. The thickness of the Stolleklint Clay is also enigmatic due to recent glacio-tectonism, but recent fine-scale mapping suggests that the thickness of the PETM ‘body phase’ is 24 ±2 m (*80*), which represents a rapid increase in sedimentation rates coincident with the start of the PETM. During the recovery phase, the Stolleklint Clay grades upwards into the diatomite-rich facies of the Fur Formation. Sedimentation rates for the Fur Formation are currently poorly constrained, but it is likely that the increased deposition of diatomite reflects a decrease in detrital inputs post-dating the PETM event.

Figure S1. Sample location map on a palaeomap from the PETM. The figure was created using the open-source plate tectonics software GPlates (*99, 100*), based on modifications to the reconstruction model (*101*) and plotted with Generic Mapping Tools (*102*). Palaeo-shorelines and marine seaways are from Golonka (2009)(*103*). See text for location details.

2. Data tables

Leg/Exp	Site	Hole	Core	Section	Interval (cm)	Depth (mbsf)	δ^7Li ‰	2sd	Li/Ca *µmol/mol*	Mg/Ca *mmol/mol*	Al/Ca *mmol/mol*	Mn/Ca *mmol/mol*	Sr/Ca *mmol/mol*	Rb/Ca *µmol/mol*	Li/(Mg+Ca) *µmol/mol*	Sr/(Mg+Ca) *mmol/mol*
Site 865 (bulk carbonates)																
143	865	C	12	1	100–110	99.3	20.0	0.3	16.0	27.3	0.08	0.27	1.02		15.6	1.0
143	865	C	12	1	130–140	99.6	19.5	0.4	13.1	18.8	0.10	0.23	0.97		12.8	0.9
143	865	C	12	2	40–50	100.2	18.6	0.2	20.0	37.4	0.11	0.25	0.94		19.3	0.9
143	865	C	12	3	40–50	101.7	18.1	0.1	18.6	24.7	0.08	0.26	0.83		18.8	0.9
rpt							17.7	0.1	18.9	26.4	0.20	0.18	0.89		18.1	0.8
143	865	C	12	3	80–90	102.1	18.1	0.4	15.7	25.2	0.10	0.20	0.88		15.5	0.9
rpt							17.5	0.3	16.0	25.9	0.06	0.30	0.84		15.6	0.8
143	865	C	12	3	120–130	102.5	18.4	0.3	17.7	36.8	0.07	0.35	0.84		17.1	0.8
143	865	C	12	4	0–10	102.8	19.9	0.4	21.6	42.1	0.11	0.38	0.63		20.7	0.6
rpt							19.9	0.2	18.7	37.1	0.06	0.41	0.58		18.1	0.6
143	865	C	12	4	40–50	103.2	19.7	0.4	16.7	30.3	0.47	0.23	0.89		16.2	0.9
143	865	C	12	4	120–130	104.0	20.2	0.1	17.9	22.6	0.07	0.21	0.86		17.6	0.8
143	865	C	12	5	40–50	104.7	20.2	0.1	16.6	21.2	0.09	0.22	0.85		16.3	0.8
143	865	C	12	5	120–130	105.5	20.7	0.6	12.4	19.9	0.17	0.19	0.82		12.2	0.8
143	865	C	13	1	20–30	108.0	19.5	0.1	24.0	25.5	0.07	0.21	0.90		23.4	0.9
143	865	C	13	1	100–110	108.8	20.4	0.3	26.3	25.5	0.10	0.28	1.10		26.7	1.1
rpt							20.4	0.2	18.3	25.6	0.07	0.22	0.86		18.0	0.9
Site 1051 (bulk carbonates)																
171	1051	B	59	3	87–88	510.18	18.4	0.4	38.5	2.2	0.03	1.88	2.5	1.05	38.5	2.5
171	1051	B	59	3	118–119	510.49	18.2	0.2	33.6	0.3	0.08	0.13	0.003	1.04	33.6	0.0
171	1051	B	60	1	63–64	511.44	18.0	0.2	21.1	1.2	0.08	0.62	1.01	0.96	21.1	1.0
171	1051	B	60	1	77–78	511.68	17.0	0.3	36.9	1.5	0.67	1.50	1.96	1.45	36.9	2.0

171	1051	B	60	1	103–104	511.84	17.2	0.4	31.1	1.6	0.37	1.21	1.7	1.12	31.1	1.7
171	1051	B	60	1	123–124	512.135	17.5	0.2	13.6	0.9	0.17	0.61	0.92	0.74	13.6	0.9
171	1051	B	60	1	142–143	512.24	18.2	0.4	23.1	1.2	0.06	0.33	1.12	1.02	23.1	1.1
171	1051	B	60	2	3–4	512.44	18.0	0.2	39.6	1.6	0.19	0.48	2.59	1.23	39.6	2.6
171	1051	B	60	2	17–18	512.58	18.2	0.1	24.5	1.9	0.18	0.63	2.23	1.83	24.6	2.2
171	1051	B	60	2	25–26	512.655	18.8	0.3	22.8	2.3	0.22	0.83	1.26	1.69	22.8	1.3
171	1051	B	60	2	40–41	512.81	20.6	0.4	39.5	1.2	0.05	0.43	1.50	0.77	39.5	1.5
171	1051	B	60	2	49–50	512.9	20.7	0.3	27.9	2.8	0.19	0.87	1.89	1.68	27.9	1.9
rpt							20.1	0.2	21.3	2.5	0.05	0.54	1.73	0.63	21.3	1.7
171	1051	B	60	2	55–56	512.96	20.7	0.1	25.2	2.7	0.20	0.42	2.54	1.51	25.2	2.5
Site 1210 (bulk foraminifera)																
198	1210	A	20	6	19–20	184.59	19.3	0.1	23.0	1.5	0.34	0.19	2.16	0.67	23.0	2.2
198	1210	A	20	6	25–26	184.65	18.9	0.1	4.7	0.23	0.08	0.03	0.38	0.10	4.7	0.4
198	1210	A	20	6	31–32	184.71	19.2	0.3	8.4	0.33	0.27	0.09	0.56	0.16	8.4	0.6
198	1210	A	20	6	40–42	184.8	18.6	0.5	21.6	0.84	0.49	0.21	1.22	0.51	21.6	1.2
198	1210	A	20	6	41–43	184.81	18.9	0.5	3.1	0.86	0.36	0.14	1.19	0.41	3.1	1.2
rpt cleaned forams							18.2	0.1	3.3	0.13	0.20	0.02	0.19	0.00	3.3	0.2
198	1210	A	20	6	43–45	184.83	19.3	0.2	5.7	0.80	0.23	0.11	1.09	0.34	5.7	1.1
198	1210	A	20	6	44–46	184.84	18.8	0.3	9.0	0.82	0.39	0.17	1.27	0.30	9.0	*1.3*
198	1210	A	20	6	45–47	184.85	18.2	0.3	2.6	0.87	0.29	0.17	1.43	0.69	2.6	1.4
198	1210	A	20	6	46–48	184.86	17.5	0.1	7.0	0.48	0.21	0.16	0.98	0.27	7.0	1.0
198	1210	A	20	6	47–49	184.87	17.8	0.3	2.5	0.56	0.47	0.16	0.74	0.28	2.5	0.7
198	1210	A	20	6	48–50	184.88	17.8	0.4	7.6	1.81	0.12	1.99	2.66	1.57	7.6	4.9
rpt cleaned forams							17.3	0.4	7.6	2.28	0.56	0.46	3.68	1.02	7.5	3.7
198	1210	A	20	6	50–51	184.9	18.0	0.2	3.6	2.64	0.10	0.57	4.95	1.23	3.6	2.7
rpt cleaned forams							18.5	0.6	4.5	2.42	0.36	0.26	0.67	0.15	4.5	0.7
198	1210	A	20	6	51–52	184.91	18.9	0.5	36.9	2.40	0.33	0.32	3.39	1.66	36.8	3.4
198	1210	A	20	6	52–54	184.92	20.2	0.6	12.0	0.57	0.48	0.13	1.20	0.25	12.0	1.2

198	1210	A	20	6	53–55	184.93	21.7	0.2	3.9	0.21	0.21	0.03	0.31	0.15	3.9	0.3
198	1210	A	20	6	55–56	184.95	21.0	0.3	41.4	0.59	0.51	0.24	1.13	0.47	41.4	1.1
198	1210	A	20	6	62–63	185.02	21.2	0.6	44.7	2.29	0.37	0.47	3.59	2.09	44.6	3.6

Table S1. Li isotope and trace element data from the marine carbonate sections. The analytical uncertainty represents the 2sd of the three repeats of each sample run during each session (see Methods). Full procedural replicates are indicated by rpt.

Sample Name	Depth	δ^7Li	2sd
Fur, Denmark			
SK17 -0.20 to -0.21	-24.76	-0.1	0.2
SK17 -0.10 to -0.11	-24.66	0.0	0.2
SK17 -0.05 to -0.06	-24.61	-0.7	0.1
SK17 -0.03 to -0.04	-24.59	-1.0	0.1
SK17 -0.01 to -0.02	-24.57	-0.4	0.4
SK17 0.11 to 0.12	-24.44	-0.2	0.4
SK17 0.18 to 0.19	-24.37	0.2	0.4
SK17 0.292 to 0.300	-24.26	-3.7	0.4
SK17B 3.5	-22.58	-2.9	0.3
SK17B 10	-17.95	-2.3	0.3
SK17B 16	-14.17	-1.9	0.4
SK17B 22	-10.48	-1.6	0.2
SK17B 25	-8.56	-1.7	0.1
SK16-B225	-2.25	-1.8	0.3
FQ16-43	-1.39	-0.7	0.2
SK16-B17	-0.17	-0.7	0.3
FQ16-76	1.71	-1.7	0.3
FQ16-78	1.91	0.2	0.3
Fur15-11.1	11.10	-0.3	0.2
Fur15-12.4	12.40	0.5	0.3
Svalbard			
BH 09/05 553.87	553.87	-0.1	0.2
BH 09/05 552.43	552.43	-0.3	0.4
BH 09/05 551.43	551.43	-0.5	0.3
BH 09/05 550.41	550.41	-0.3	0.3
BH 09/05 540.42	540.42	-0.4	0.1
BH 09/05 537.03	537.03	-1.4	0.1
BH 09/05 536.33	536.33	-1.3	0.1
BH 09/05 533.36	533.36	-1.3	0.1
BH 09/05 531.03	531.03	-2.3	0.3
BH 09/05 528.08	528.08	-2.3	0.3
BH 09/05 521.05	521.05	-0.7	0.3
BH 09/05 512.03	512.03	-0.3	0.4
BH 09/05 477.03	477.03	-0.6	0.3
BH 09/05 471.55	471.55	-0.8	0.4

BH 09/05 439.93	439.93	-0.4	0.3

Table S2. Li isotope data from the shale sections.

3. Trace element ratios of carbonates

Li/Ca ratios of the carbonates show a similar range for all sections (~3–45 μmol/mol), with maximum values similar to modern core-top carbonates (Fig. S4)(*7*). There is no obvious trend in Li/Ca ratios across the PETM for any of the sections (Fig. S2), but a trend would not necessarily be expected, given regional temperature and salinity effects (*6, 104*), as well as changing seawater Li concentrations. Similar stability was found for Li/Ca ratios from Ocean Anoxic Events 1a and 2 (*42, 45*). Mn/Ca ratios generally exhibit a slight peak at the PETM, although absolute values vary between cores, and such variability likely reflects changing redox conditions during this time period (*105*). There is no correlation between Mn/Ca and δ^7Li (Fig. S3), indicating no effect from Mn-oxyhydroxides. On the note of oxyhydroxides, Site 1210 has also been investigated for Cr isotopes, which would be controlled by such minerals if strongly present (*106*). In all cases, Al/Ca ratios are low, indicating that the effect of silicate dissolution during sample leaching is insignificant (Table S1). All Al/Ca values are below the cutoff used in prior reconstructions of seawater Li isotope ratios from bulk carbonates (*42*), while most are also beneath the lower cutoff recently suggested in a study specifically examining at the effects of diagenesis on Li isotope ratios in carbonates (*29*). Where Rb/Ca ratios are available (Table S1), no relationships are seen with δ^7Li, again suggesting minimal silicate contamination (since Rb is expected to be derived from silicates). At Site 1210, elemental ratios from the leaching method and by the traditional foraminiferal cleaning method are similar, and critically Al/Ca is similar, implying that very weak bulk leaching is a viable method for recovering Li signatures from foraminifera. Potential diagenetic effects on observed δ^7Li values are discussed in the main text.

Finally, there is no correlation observed at Site 1210 between foraminiferal δ^7Li and diversity indices of planktonic foraminifera (*107*), suggesting that changing species across the PETM are not resolvably affecting δ^7Li.

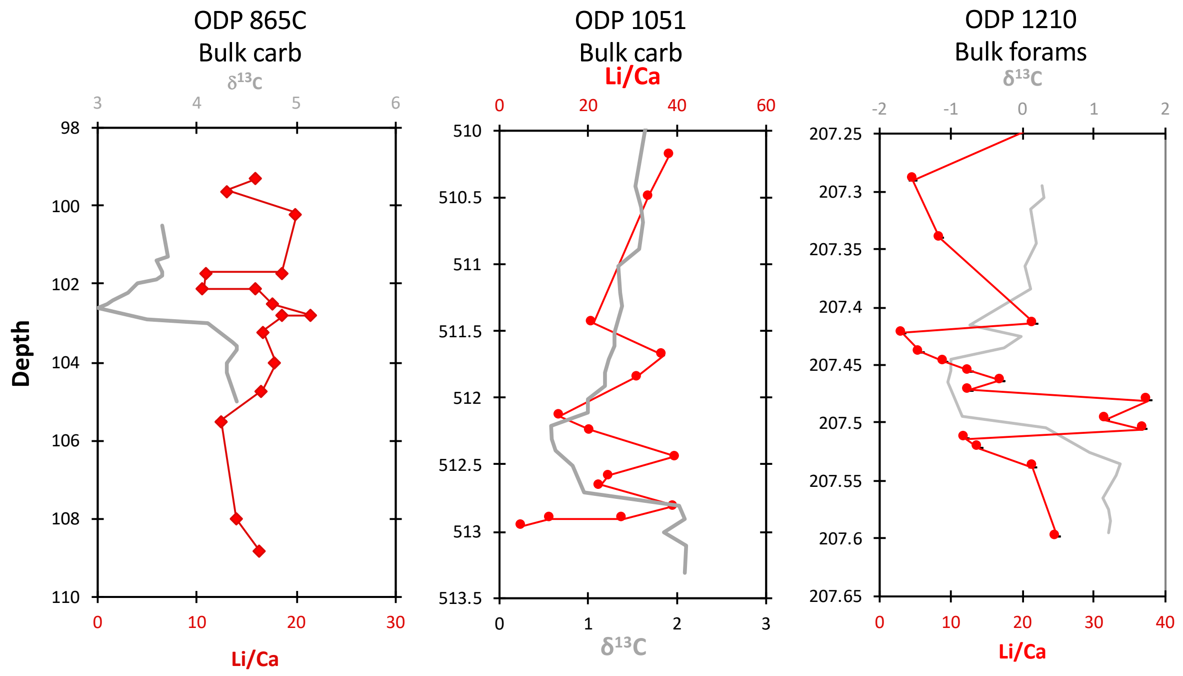

Figure S2. Carbonate Li/Ca ratios for each section.

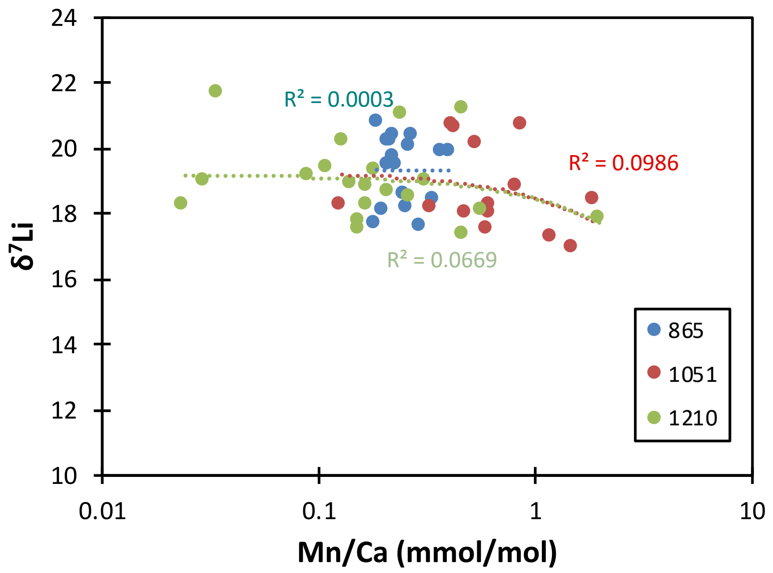

Figure S3. Lack of relationship between Mn/Ca and δ^7Li for the three carbonate sections, indicating no identifiable effect of Mn-oxyhydroxides on Li isotope ratios.

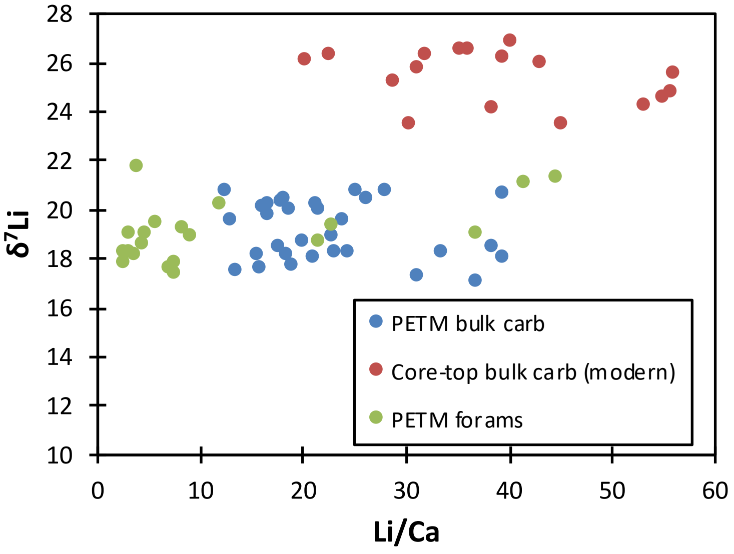

Figure S4. Bulk carbonate and foraminifera Li/Ca vs. δ^7Li from this study, compared to modern core-tops (*7, 29*).

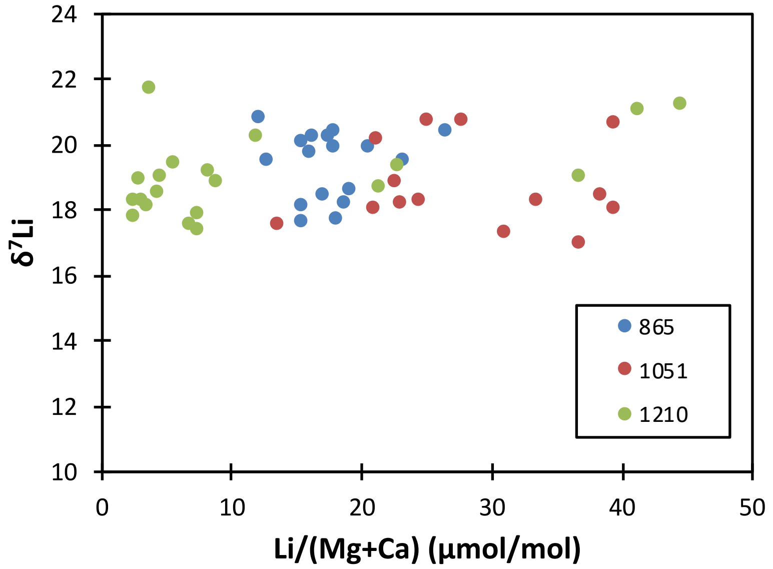

Figure S5. Comparison of Li/(Mg+Ca) and δ^7Li values of bulk carbonates (ODP 865 and 1051) and foraminifera (ODP 1210) from this study. See the main text for discussion on diagenesis in the context of recent work on these effects (*29*).

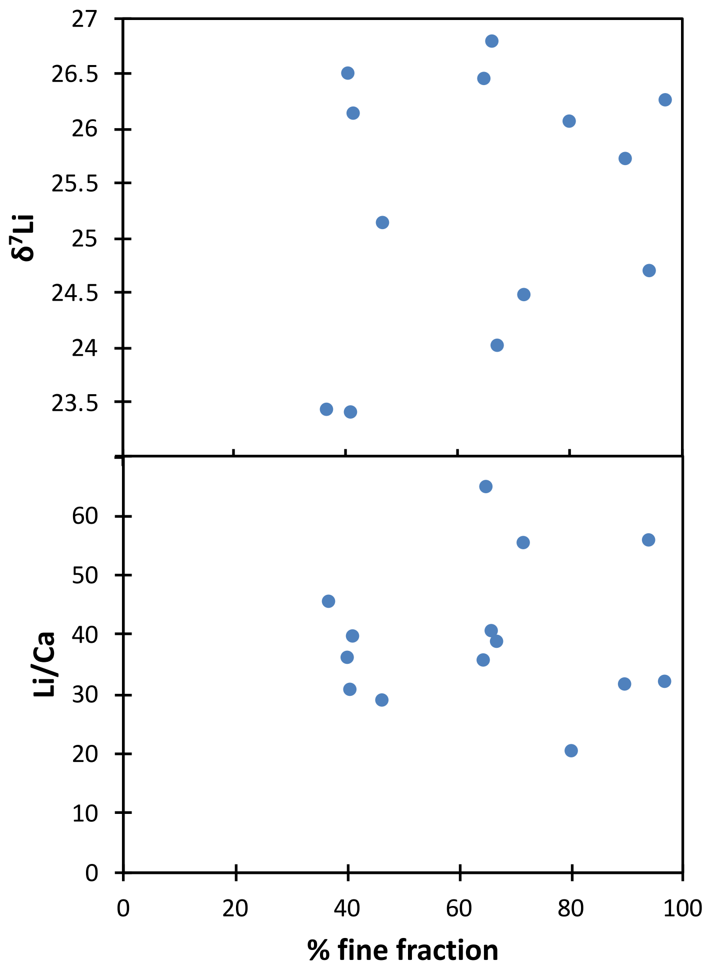

Figure S6. Results from modern core-tops (*7*), showing no relationship between the fine fraction (indicating proportion of coccolithophores vs. foraminifera) and δ^7Li or Li/Ca.

4. Timing of the Li isotope excursion

At Site 865, using the age model from (*41*), the time from onset to maximum Li isotope excursion (minimum δ^7Li) is ~202 kyr. At Site 1051, using the age model of (*84*), the duration is ~180 ± 15 kyr. Both durations are the same given the sampling resolution of these cores.

In contrast, the duration at Site 1210 is 64 kyr using the age model of (*87*), or 105 kyr using the age model of (*85*). The oceanic residence time of Li is on the order of a million years (*108*), and therefore the oceans are isotopically homogenous at any given time. This finding therefore raises concerns about the completeness of the age model for Site 1210, in particular for the recovery phase of the CIE. Overall, this concern is supported by the dynamic box models of Li discussed below (Section 7), where it is very hard to force an oceanic recovery of Li in less than ~200 kyr.

5. Ash dissolution as a potential influence on the shale sections

A significant volcanic episode prior to or coincident with the PETM was identified in Svalbard (*40*), and calculated the amount of dissolved ash required to have caused their observed unradiogenic osmium isotope excursion in this basin. Combining this estimate with the Li concentrations and isotope composition of ash from the 2010 Eyjafjallajökull eruption in Iceland (*109*), which is basaltic and sourced from the same mantle plume as the ash at the PETM, approximately 10^{11}g of Li would have been dissolved into the oceans in the first 8000 years of the PETM. This source of Li is about 3 orders of magnitude smaller than the Li source inferred from modelled weathering rate increases over the first 8000 years (*3, 5*), or from the models in this study (Section 7). Hence, dissolution of volcanic ash could not have contributed significantly to the global seawater Li isotope excursion. This conclusion is also supported by observations on a smaller scale in the Fur section, where ash layers exist both before and during the Li isotope excursion, but do not directly coincide with any of the major δ^7Li shifts recorded in the shales (Fig. S7), indicating no resolvable effect of the ash on the composition of local Li inputs.

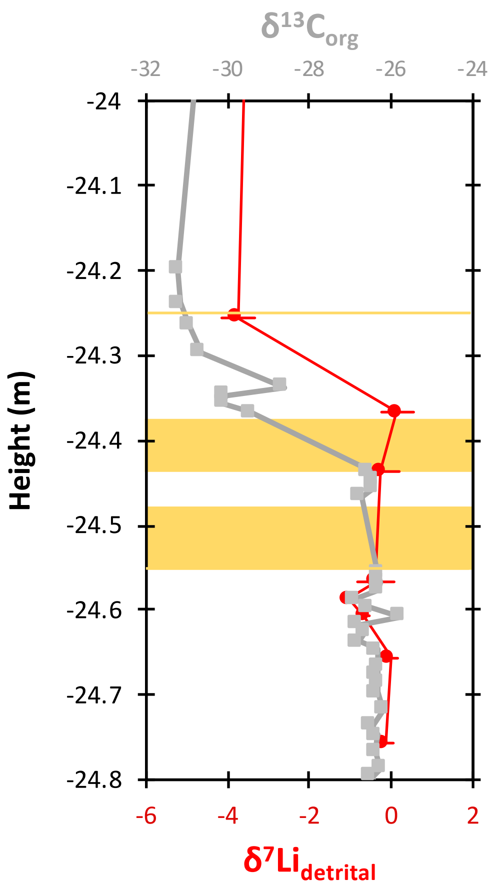

Figure S7. An expanded section showing the initiation of the PETM in the Fur section. The yellow bands represent ash layers (*80*).

6. Details of modern Li isotope behaviour

Lithium isotopes have been shown not to be fractionated by plant growth or primary productivity (*56, 110, 111*) and are not sensitive to carbonate weathering (*112, 113*), given the low Li content of carbonates. Although evaporite weathering can be locally significant (*114*), on a global scale the evaporite contribution is relatively minor in modern rivers (*20*). The δ^7Li of primary silicate rocks defines a narrow range (continental crust ~0.6 ± 0.6 ‰, basalt ~3–5 ‰ (*54, 115*)) compared to the high variability of dissolved Li in modern rivers (2–44 ‰)(*22, 23, 25, 114, 116-118*). Therefore, fluvial values overwhelmingly reflect weathering processes (*37, 119-122*) , and in particular the relative importance of rock dissolution (driving riverine values to low, rock-like, δ^7Li compositions) to secondary mineral formation (driving riverine values to high δ^7Li)(*118*). This ratio is generally known as the weathering congruency (*23, 24, 118*). As a result, lithium isotopes can also inform on

weathering intensity, that is the ratio of weathering rates to denudation rates (W/D)(*25*), where the denudation rate is the sum of the chemical weathering rate and the physical erosion rate. Weathering intensity is tied closely to congruency. Low intensity weathering (low W/D) results in low riverine δ^7Li; as weathering intensity increases, clay formation and riverine δ^7Li also increase (Fig. 5, main text). Very high intensity regimes also exist, where δ^7Li is low due to the redissolution of (or desorption from) pre-formed secondary clays. These regimes only exist in strongly supply-limited areas, such as tropical lowlands. Overall, both the riverine Li yield and flux decrease by almost two orders of magnitude from low to high intensity regimes (Fig. 5b, c). Hence, globally-pervasive high-intensity weathering would have very little influence on the oceans' Li budget, which would then be dominated by the hydrothermal input (*23, 25*). In the modern oceans, ~60 % of the Li inputs are via rivers with a mean δ^7Li ~23 ‰, and ~40 % is via mid-ocean ridge hydrothermal solutions with a mean δ^7Li ~7 ‰ (*27*). Lithium is predominantly removed by incorporation into low-temperature clays, both in altered oceanic crust (AOC) and marine aluminous authigenic clays (MAAC). These clays both preferentially remove light Li and together these sinks impose an isotopic fractionation of ~15 ‰, which drives modern seawater higher to 31 ‰ (*123, 124*). Marine carbonates represent a negligible sink for Li, but have frequently been used as an archive for past seawater Li isotope ratios in order to constrain past silicate weathering behaviour (*8, 24, 27, 34, 42, 45, 125*). Given that the global ocean residence time for Li of ~1 million years (*126, 127*) is significantly longer than the ocean mixing time, we anticipate agreement between Li isotope reconstructions from each location if the composition of palaeo-seawater is being recorded.

The references used for Figure 5 are: Orinoco (*116, 128*), Mackenzie (*113*), Changjiang (*129*), Amazon (*25*), Yangtze (*114*), Siberia (*128*), Iceland (*130*), Congo (*131*), Lena (*22*), Azores (*132*). Overall, the figure is modified from refs (*8, 25*).

7. Dynamic box models

While there is no direct correlation between silicate weathering rates or fluxes and riverine Li isotope ratio s(*23*), it is possible to reconstruct changes in weathering by modelling the observed seawater isotope ratios (*34, 42, 45*). Here we use a dynamic ocean box model to constrain possible mechanisms for changing both seawater Li concentration

and isotope ratio. This model, in different degrees of complexity, is well-described in several other publications (*34, 42, 45*).

It is theoretically possible to reproduce the observed changes in seawater δ^7Li by only changing the hydrothermal input for 100 kyrs by 3.2× relative to pre-excursion values (Fig. S8), or for a 200 kyr excursion with a 2.2× increase. However, such a change in hydrothermal input is significantly greater than has been reported for any time during the Cenozoic, and is therefore discounted in our interpretation.

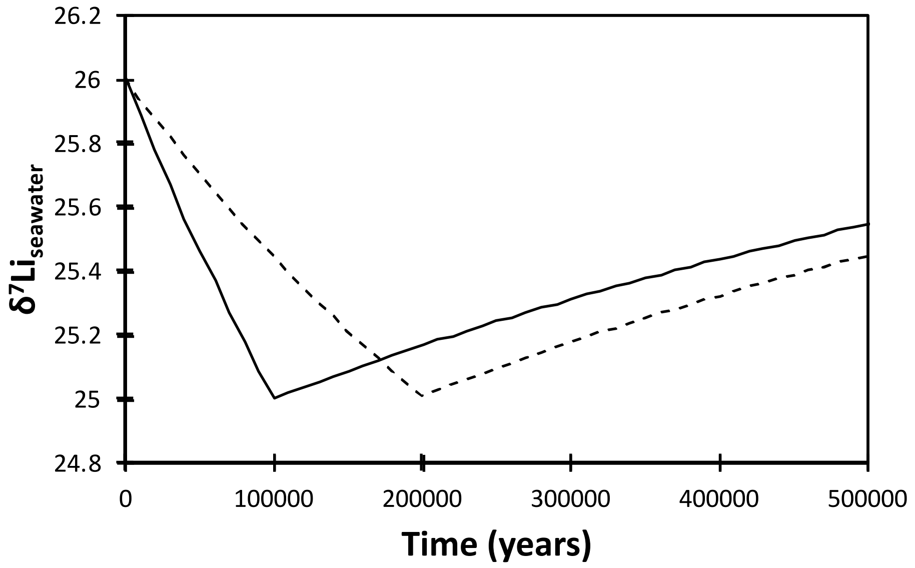

Figure S8. Model showing seawater δ^7Li if an increase in the hydrothermal flux were solely responsible for the observed excursion. A 1 ‰ negative excursion requires a 2.2× increase for 200 kyr (dotted line), or a 3.2× increase for 100 kyr (solid line).

Assuming that the hydrothermal input can be constrained from mid-ocean ridge spreading rates, the PETM hydrothermal input is between 1.15× and 1.4× that of the present (*46, 47, 133*). The maximum effect of this range in hydrothermal input to steady-state seawater δ^7Li is a shift of 0.5 ‰. In the model, we apply an intermediate value of 1.25× that we maintain constant throughout the PETM onset, peak, and recovery. There are then effectively two unknowns: the riverine Li flux and the riverine Li isotope ratio. A change in the flux with no change in riverine δ^7Li would change seawater δ^7Li, as would a change in riverine δ^7Li under constant flux conditions. It is also possible for both flux and isotope ratio to change simultaneously.

While lithium has a long oceanic residence time (1 Myr), it is still possible to resolve an excursion of less than ~100 kyr for sufficiently large weathering changes (*34, 42*). This is because the first half of the excursion is due to a major Li addition, driving a rapid seawater δ^7Li change. The subsequent recovery back to pre-excursion seawater values is strongly dependent on residence time (Fig. S9).

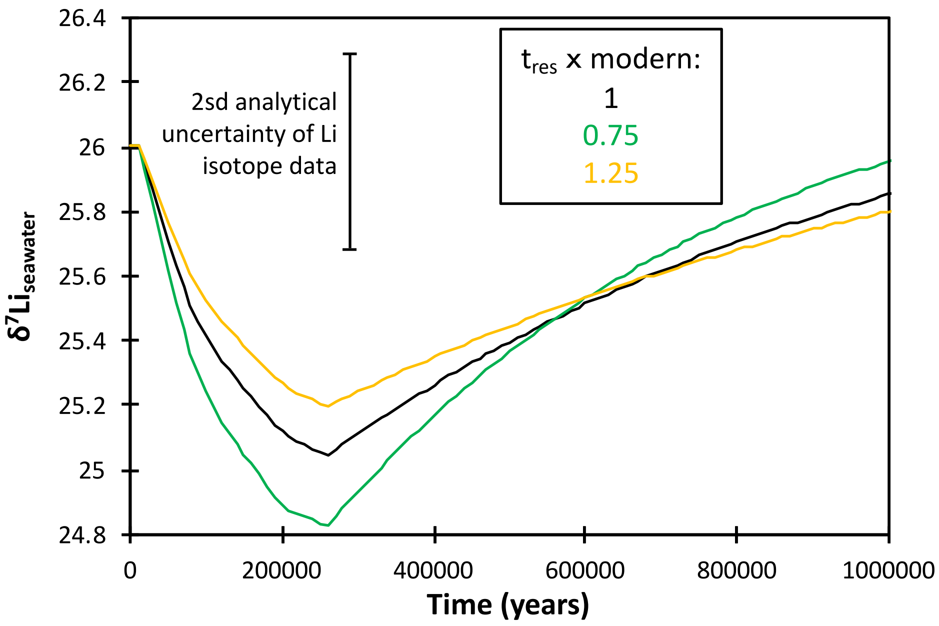

Figure S9. Effect on seawater δ^7Li of different oceanic residence times of Li (modern, used in the main model, and 0.75x and 1.25x the modern).

As described in the main text, we take the riverine Li flux as a known parameter, by mapping the silicate weathering outputs of two Earth System models onto the Li flux. One of these models is a GENIE model, while the other is a LOSCAR model (*3, 5*). Both models have distinct silicate weathering calculations.

In cGENIE, weathering is controlled by the RoKGeM model, where the weathering rate is a function of both temperature and water-rock interaction time (runoff)(*134*). In PETM reconstructions using cGENIE, models were also run with the temperature-feedback on weathering switched off (*3*). The silicate weathering rates used here were derived from the Ca flux (a combination of carbonate and silicate weathering) and the DIC flux (which stems from carbonate weathering and volcanic CO_2 outgassing), calculated by (*3*). By substituting, we gain the silicate weathering flux directly (Ridgwell, personal communication).

In LOSCAR, carbonate and silicate weathering fluxes are direct outputs. The silicate weathering rate is parameterised as a function of pCO_2. Importantly, the strength of the weathering feedback and the initial (steady-state) weathering flux are also controlled by the model (*49, 50*). This allowed for initial testing of the feedback strength for the PETM in the original model (*5, 9*), which in turn effectively allows for parameterisation of regolith thickness.

Both models suggest an initial (steady-state) weathering flux similar to the present day, which also agrees with scenarios suggested by Li and West (2014)(*36*). However, some other models (*47*) have suggested that the global weathering flux was low prior to the PETM, because of enhanced soil cover relative to the present day. We test such a scenario by applying an initial riverine weathering flux that is 60% of the modern flux (*36, 47*). However, in this case, the riverine flux is then low compared to the hydrothermal flux (*36, 46, 133*), which results in a weaker control on seawater δ^7Li by river δ^7Li. Therefore, even decreasing δ^7Li of rivers to the lowest possible value (0‰, with a decrease from pre-PETM values of 17‰) only results in a 0.5‰ negative seawater excursion, which is not sufficient to explain the observed data. In other words, the observed data cannot be modelled by starting with a low riverine weathering flux (Fig. S10).

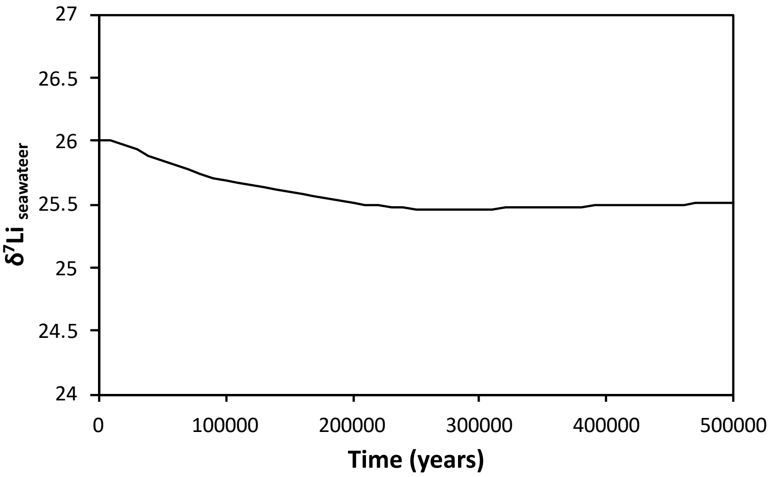

Figure S10. Model for a low initial riverine flux and a 17‰ decrease in riverine δ^7Li during the PETM (using a modern seawater Li budget). A low initial river flux cannot create a large enough negative excursion to explain the observed data.

Equally, the observed excursion cannot be reproduced if the trends for modern rivers between δ^7Li, weathering intensity, δ^7Li, and dissolved Li fluxes are followed (main text Fig. 5), and the global regime evolves to a higher intensity regime (i.e. riverine δ^7Li decreases, but flux also decreases – Fig. S11).

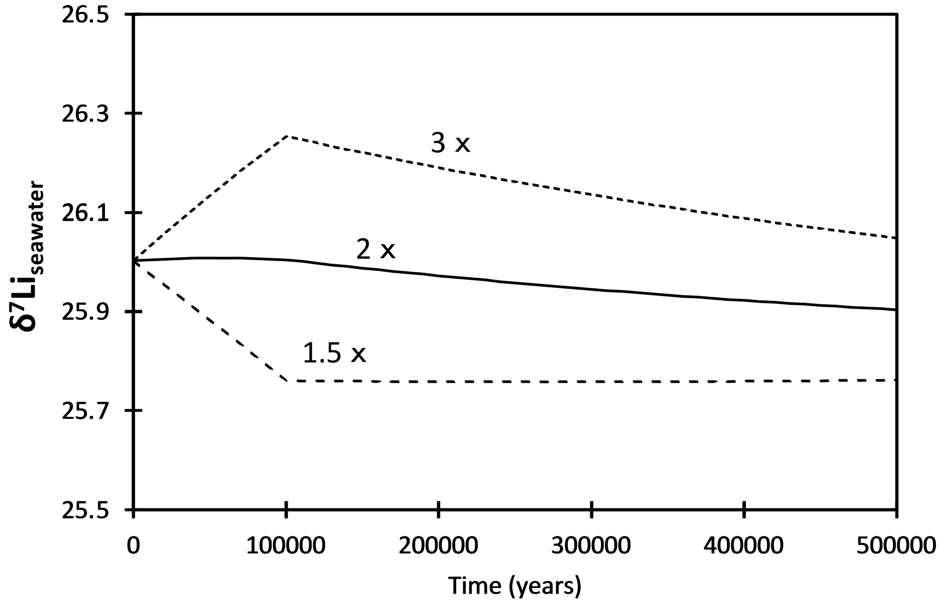

Figure S11. Models of seawater δ^7Li if the global weathering evolves to a more intense regime, following the trends from present-day rivers (main text Fig. 5). In all models, the riverine δ^7Li shifts to 0‰, and the different lines show the effect of decreasing riverine flux by 1.5, 2 and 3 times for 100 kyr. The increase in oceanic δ^7Li for a 3x decrease is due to a transient adjustment of the seawater Li budget, as also observed in other studies (*34*).

As described in the main text, an alternative scenario is for PETM rivers to have deviated from modern riverine behaviour, specifically for clay dissolution to have a high Li flux. Figure S12 shows model results for the cGENIE-derived model, if the riverine Li flux had a δ^7Li of -3.8‰, the lowest Li isotope ratio from our detrital sections. A 1‰ excursion would require a 1.1× increase in riverine flux. If this were sourced from saprolites (*135*) or laterites (*55*), then 7.5–15 Gt of clay dissolution per year would be required (Fig. S12).

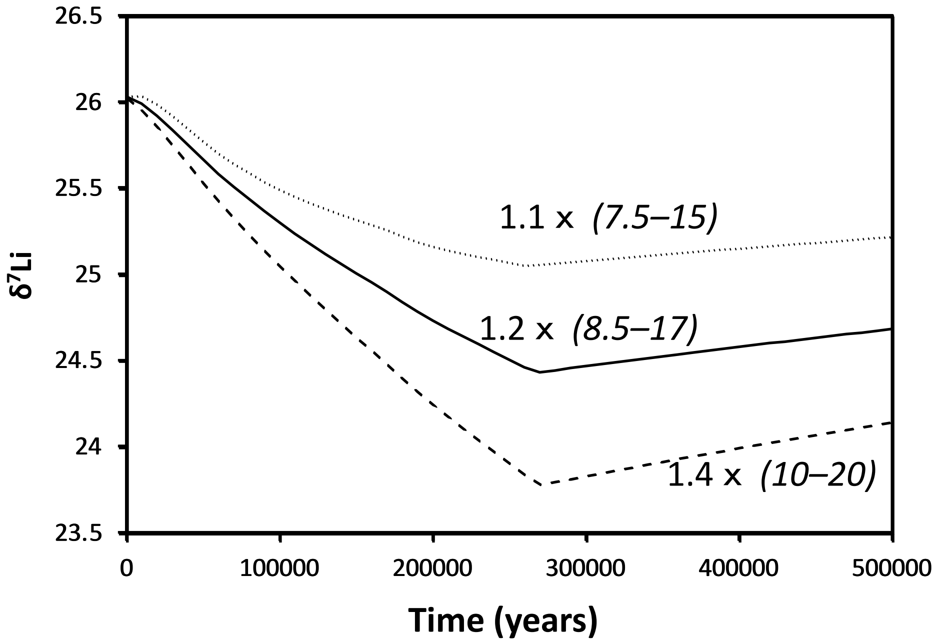

Figure S12. Models of seawater δ^7Li for an excursion is solely driven by the dissolution of clay with a δ^7Li of -3.8‰. The first number for each curve is the amount that dissolution increased by compared to pre-PETM values. The italicised numbers in parentheses indicate the amount of clay dissolved per year at peak dissolution in Gt, assuming that the clays were saprolites (~10 μg/g Li)(*135*) or laterites (~5 μg/g Li)(*55*).

In modern rivers for which the Li flux is sufficient to affect seawater compositions (i.e. where the weathering intensity is not very high; see Section 6)(*25*), Li flux and isotope composition co-vary with weathering intensity (Fig. 5, main text). We assume that a similar relationship also existed in temporal trends of the global riverine average across the PETM, as suggested for other time periods (*34, 42, 45*), and therefore scale the riverine δ^7Li composition to the modelled riverine Li flux through time. The precise gradient of this scaling varies slightly according to the model, so that the required negative δ^7Li excursion is achieved for a given weathering flux increase. We determine the magnitude of riverine δ^7Li changes required to generate a seawater Li isotope excursion of ~1 ‰ in each model.

We assess model sensitivity by applying a ±10 % uncertainty to the riverine Li flux and a ±1 ‰ uncertainty to the riverine Li isotope ratio, and propagating these uncertainties through the calculations (Fig. S13 and S14).

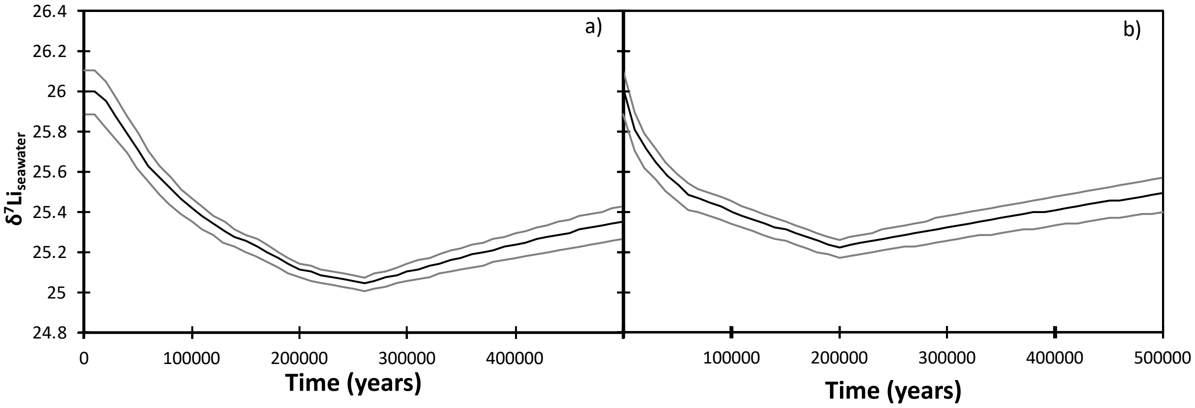

Figure S13. Effect of varying the riverine Li flux by ±10 % on seawater δ^7Li in the two model runs: (a) GENIE-based; (b) LOSCAR-based.

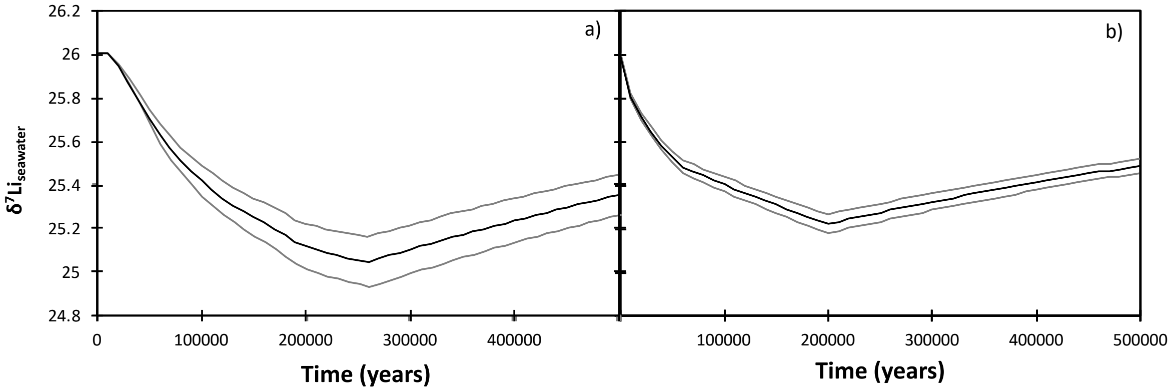

Figure S14. Effect of varying the riverine Li isotope ratio by ±1 ‰ on seawater δ^7Li in the two model runs: (a) GENIE-based; (b) LOSCAR-based. This uncertainty is effectively the uncertainty in the carbonate fractionation factor (*7*).

The increased weathering fluxes increase seawater Li concentrations by 5 % over pre-PETM values for the GENIE-derived model and by 2 % for the LOSCAR model. Such changes would not be resolvable in Li/Ca measurements, even if there were no secondary controls on that ratio in carbonates. In both cases, the peak of seawater Li concentration occurs 180–190 kyr after peak weathering is reached. This lag is attributable to an extended interval of enhanced weathering and buffering by the long residence time of Li in seawater. The change in W/D is then derived from the modern river relationship between W/D, Li yield and δ^7Li (Fig. 5). Given the 'known' increase in W from the Earth System models, we

can quantitatively calculate a corresponding increase in D, which equals the sum of W and E (the weathering and erosion rates).

8. Shale section mineralogical effects

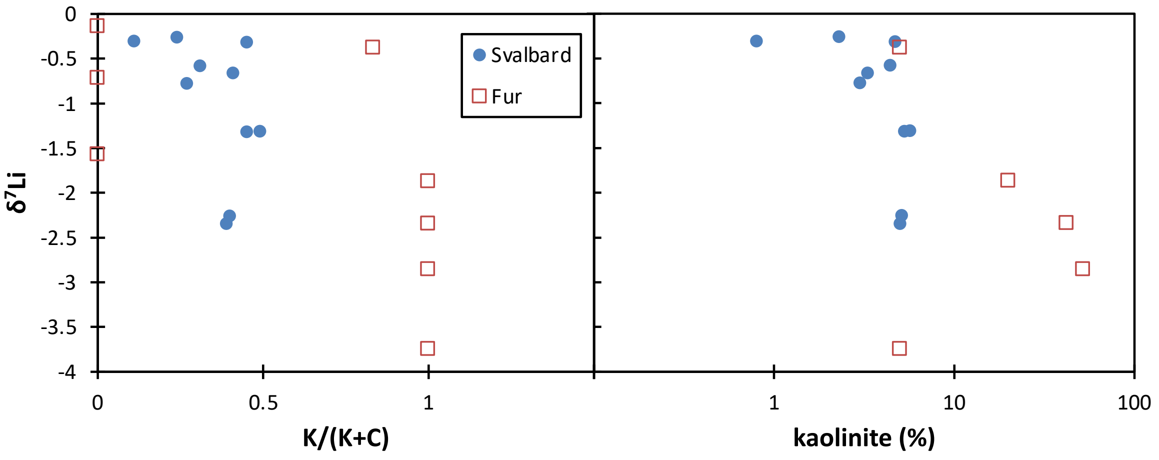

Figure S15. Lithium isotopes in detrital sediments as a function of clay mineralogy (K = kaolinite; C = chlorite) for the Svalbard and Fur sections, demonstrating no mineralogical control on Li isotopes. The correlations are not significant at the $p < 0.05$ level (Spearman Rank correlation).

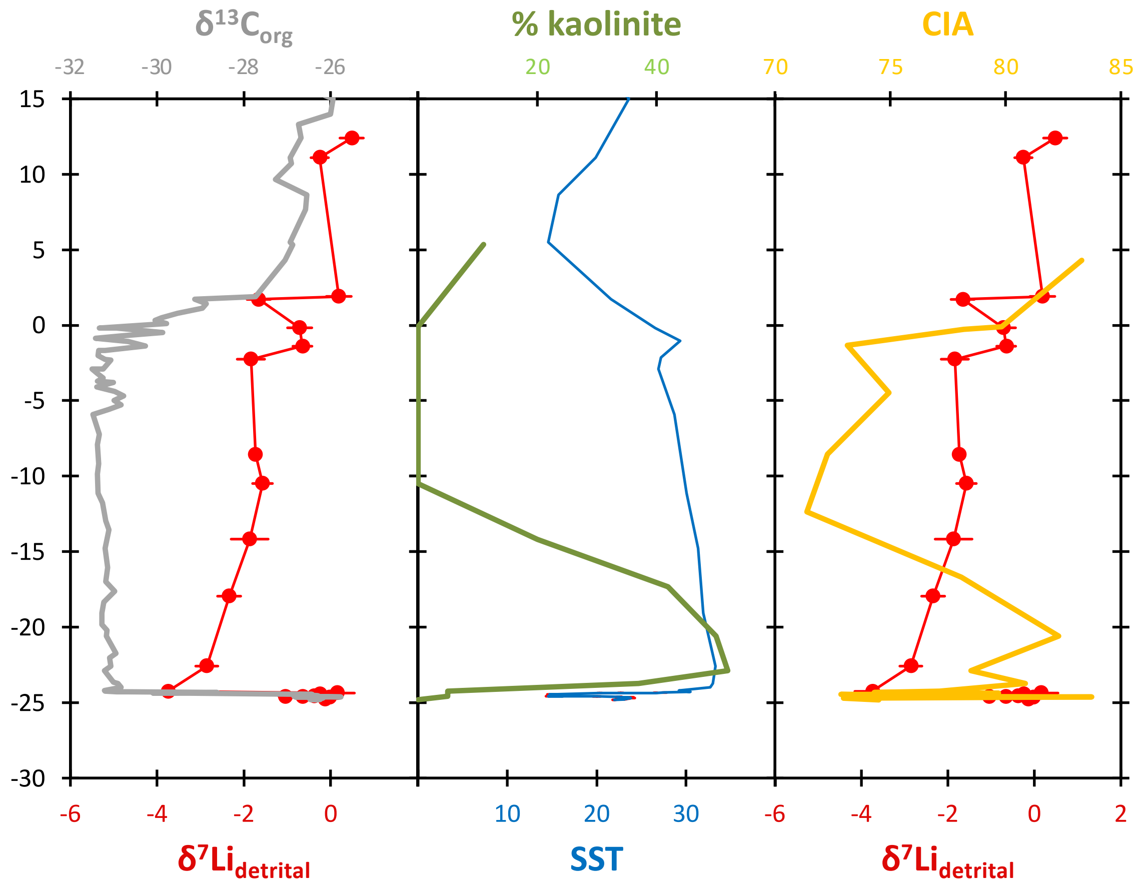

Figure S16. Comparison of the Fur section Li isotopes and $\delta^{13}C$ with the secondary clay mineralogy, the chemical index of alteration and the TEX-86 derived sea surface temperature (*19, 39*).

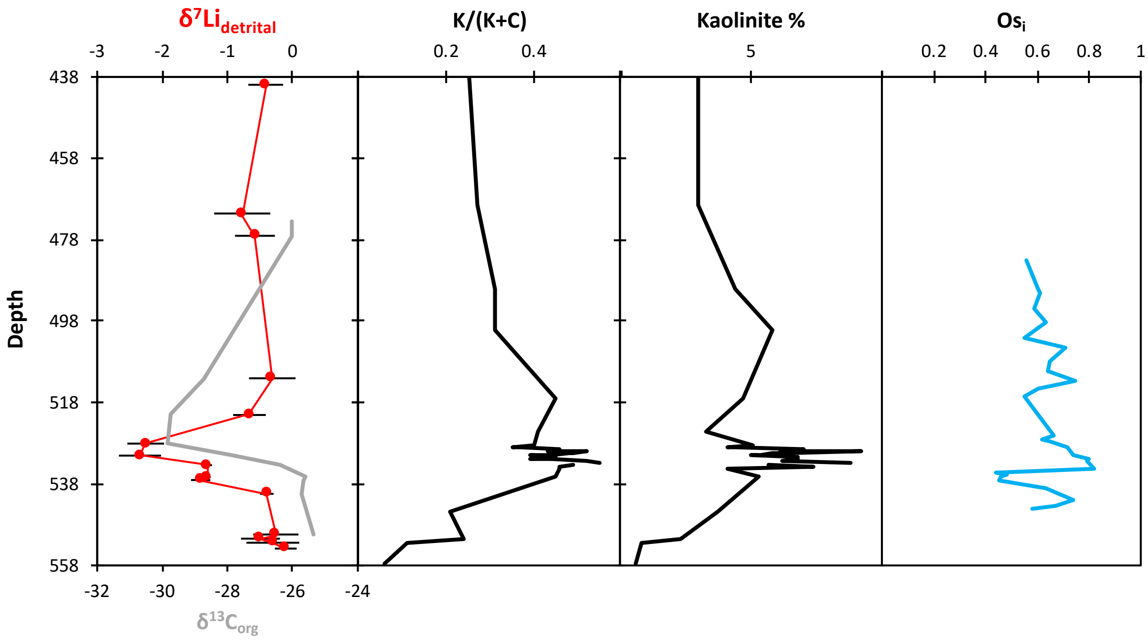

Figure S17. Comparison of the Svalbard section Li isotopes and $\delta^{13}C$ with the secondary clay mineralogy (K = kaolinite; C = chlorite) and initial osmium isotope ratios (Os_i)(*17, 40*). The results demonstrate no direct correlation with either secondary mineralogy or weathering source (see Fig. S15).

9. Modern river weathering and erosion behaviour with runoff

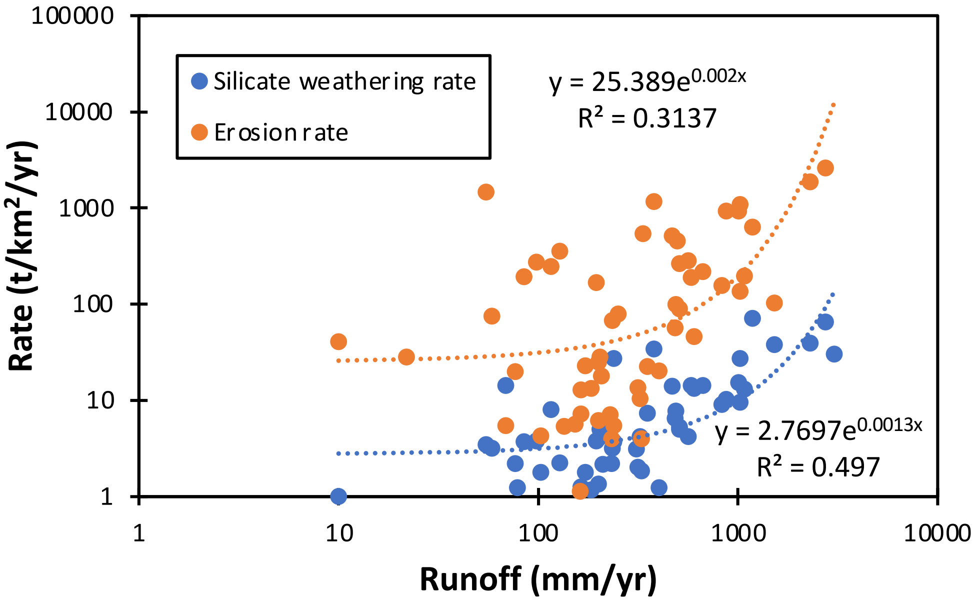

Figure S18. Silicate weathering and erosion data from the world's largest rivers with runoff (*20*). The relationships show that erosion increases faster with increasing runoff than the weathering rate, as also suggested by our PETM data.

10. Carbon burial at Site 1051

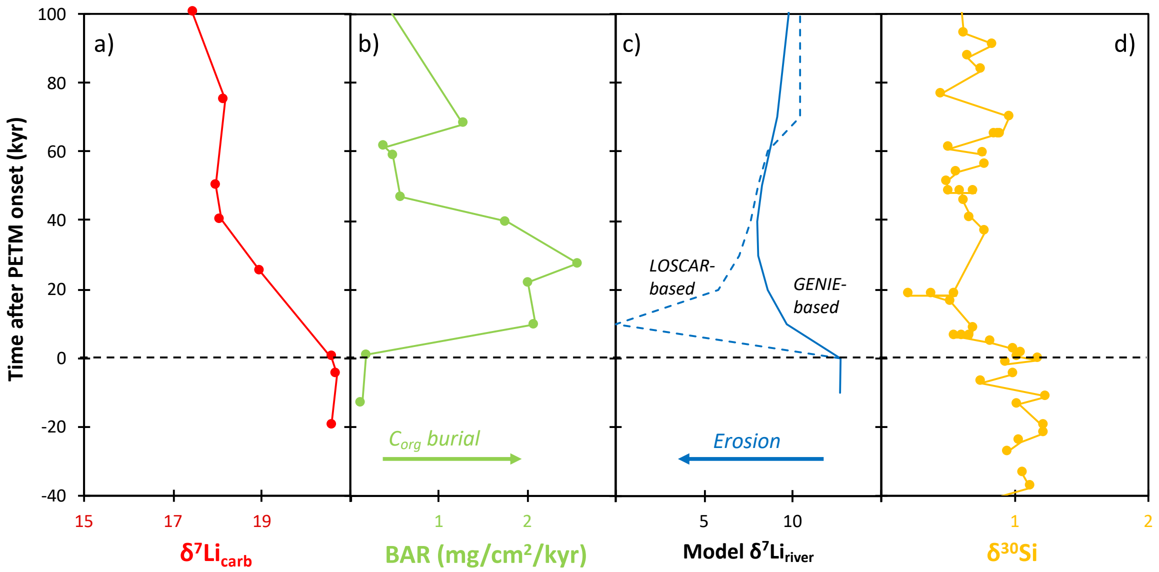

Figure S19. Comparison for Site 1051 of (a) Li isotopes, and (b) barium accumulation rates (BAR) as a proxy for organic carbon burial rates (*79*). Panel (c) shows the model results for the Li isotope composition of the global riverine input, and panel (d) shows radiolarian Si isotope ratios (*51*).

REFERENCES AND NOTES

1. M. J. Carmichael, G. N. Inglis, M. P. S. Badger, B. D. A. Naafs, L. Behrooz, S. Remmelzwaal, F. M. Monteiro, M. Rohrssen, A. Farnsworth, H. L. Buss, A. J. Dickson, P. J. Valdes, D. J. Lunt, R. D. Pancost, Hydrological and associated biogeochemical consequences of rapid global warming during the Paleocene-Eocene Thermal Maximum. *Glob. Planet. Chang.* **157**, 114–138 (2017).

2. T. D. Jones, D. J. Lunt, D. N. Schmidt, A. Ridgwell, A. Sluijs, P. J. Valdes, M. Maslin, Climate model and proxy data constraints on ocean warming across the Paleocene–Eocene Thermal Maximum. *Earth Sci. Rev.* **125**, 123–145 (2013).

3. M. Gutjahr, A. Ridgwell, P. F. Sexton, E. Anagnostou, P. N. Pearson, H. Pälike, R. D. Norris, E. Thomas, G. L. Foster, Very large release of mostly volcanic carbon during the Palaeocene–Eocene Thermal Maximum. *Nature* **548**, 573–577 (2017).

4. G. J. Bowen, J. C. Zachos, Rapid carbon sequestration at the termination of the Palaeocene–Eocene Thermal Maximum. *Nat. Geosci.* **3**, 866–869 (2010).

5. N. Komar, R. E. Zeebe, Redox-controlled carbon and phosphorus burial: A mechanism for enhanced organic carbon sequestration during the PETM. *Earth Planet. Sci. Lett.* **479**, 71–82 (2017).

6. C. S. Marriott, G. M. Henderson, R. Crompton, M. Staubwasser, S. Shaw, Effect of mineralogy, salinity, and temperature on Li/Ca and Li isotope composition of calcium carbonate. *Chem. Geol.* **212**, 5–15 (2004).

7. P. A. E. Pogge von Strandmann, D. N. Schmidt, N. J. Planavsky, G. Wei, C. L. Todd, K. H. Baumann, Assessing bulk carbonates as archives for seawater Li isotope ratios. *Chem. Geol.* **530**, 119338 (2019).

8. P. A. E. Pogge von Strandmann, S. A. Kasemann, J. B. Wimpenny, Lithium and lithium isotopes in Earth's surface cycles. *Elements* **16**, 253–258 (2020).

9. N. Komar, R. E. Zeebe, Oceanic calcium changes from enhanced weathering during the Paleocene-Eocene thermal maximum: No effect on calcium-based proxies. *Paleoceanography* **26**, PA3211 (2011).

10. K. Lalonde, A. Mucci, A. Ouellet, Y. Gelinas, Preservation of organic matter in sediments promoted by iron. *Nature* **483**, 198–200 (2012).

11. M. J. Kennedy, T. Wagner, Clay mineral continental amplifier for marine carbon sequestration in a greenhouse ocean. *Proc. Natl. Acad. Sci. U.S.A.* **108**, 9776–9781 (2011).

12. T. Dunkley Jones, H. R. Manners, M. Hoggett, S. Kirtland Turner, T. Westerhold, M. J. Leng, R. D. Pancost, A. Ridgwell, L. Alegret, R. Duller, S. T. Grimes, Dynamics of sediment flux to a bathyal continental margin section through the Paleocene–Eocene Thermal Maximum. *Clim. Past* **14**, 1035–1049 (2018).

13. G. Ravizza, R. N. Norris, J. Blusztajn, M.-P. Aubry, An osmium isotope excursion associated with the late Paleocene Thermal Maximum: Evidence of intensified chemical weathering. *Paleoceanography* **16**, 155–163 (2001).

14. B. Z. Foreman, P. L. Heller, M. T. Clementz, Fluvial response to abrupt global warming at the Palaeocene/Eocene boundary. *Nature* **491**, 92–95 (2012).

15. L. Handley, A. O'Hollaran, P. N. Pearson, E. Hawkins, C. J. Nicholas, S. Schouten, I. K. McMillan, R. D. Pancost, Changes in the hydrological cycle in tropical East Africa during the Paleocene–Eocene Thermal Maximum. *Palaeogeogr. Palaeoclimatol. Palaeoecol.* **329–330**, 10–21 (2012).

16. A. Bornemann, R. D. Norris, J. A. Lyman, S. D'haenens, J. Groeneveld, U. Röhl, K. A. Farley, R. P. Speijer, Persistent environmental change after the Paleocene–Eocene Thermal Maximum in the eastern North Atlantic. *Earth Planet. Sci. Lett.* **394**, 70–81 (2014).

17. H. Dypvik, L. Riber, F. Burca, D. Rüther, D. Jargvoll, J. Nagy, M. Jochmann, The Paleocene–Eocene thermal maximum (PETM) in Svalbard—Clay mineral and geochemical signals. *Palaeogeogr. Palaeoclimatol. Palaeoecol.* **302**, 156–169 (2011).

18. C. M. John, N. R. Banerjee, F. J. Longstaffe, C. Sica, K. R. Law, J. C. Zachos, Clay assemblage and oxygen isotopic constraints on the weathering response to the Paleocene-Eocene thermal maximum, east coast of North America. *Geology* **40**, 591–594 (2012).

19. E. W. Stokke, M. T. Jones, L. Riber, H. Haflidason, I. Midtkandal, B. P. Schultz, H. H. Svensen, Rapid and sustained environmental responses to global warming: The Paleocene–Eocene Thermal Maximum in the eastern North Sea. *Clim. Past Discuss.* (2020).

20. J. Gaillardet, B. Dupre, P. Louvat, C. J. Allegre, Global silicate weathering and CO_2 consumption rates deduced from the chemistry of large rivers. *Chem. Geol.* **159**, 3–30 (1999).

21. A. J. West, A. Galy, M. Bickle, Tectonic and climatic controls on silicate weathering. *Earth Planet. Sci. Lett.* **235**, 211–228 (2005).

22. M. J. Murphy, D. Porcelli, P. A. E. Pogge von Strandmann, C. A. Hirst, L. Kutscher, J. A. Katchinoff, C. M. Mörth, T. Maximov, P. S. Andersson, Tracing silicate weathering processes in the permafrost-dominated Lena River watershed using lithium isotopes. *Geochim. Cosmochim. Acta* **245**, 154–171 (2019).

23. P. A. E. Pogge von Strandmann, P. J. Frings, M. J. Murphy, Lithium isotope behaviour during weathering in the Ganges Alluvial Plain. *Geochim. Cosmochim. Acta* **198**, 17–31 (2017).

24. S. Misra, P. N. Froelich, Lithium isotope history of cenozoic seawater: Changes in silicate weathering and reverse weathering. *Science* **335**, 818–823 (2012).

25. M. Dellinger, J. Gaillardet, J. Bouchez, D. Calmels, P. Louvat, A. Dosseto, C. Gorge, L. Alanoca, L. Maurice, Riverine Li isotope fractionation in the Amazon River basin controlled by the weathering regimes. *Geochim. Cosmochim. Acta* **164**, 71–93 (2015).

26. J. D. Milliman, J. P. M. Syvitski, Geomorphic/tectonic control of sediment discharge to the ocean: The importance of small mountainous rivers. *J. Geol.* **100**, 525–544 (1992).

27. E. C. Hathorne, R. H. James, Temporal record of lithium in seawater: A tracer for silicate weathering? *Earth Planet. Sci. Lett.* **246**, 393–406 (2006).

28. D. Vance, D. A. H. Teagle, G. L. Foster, Variable quaternary chemical weathering fluxes and imbalances in marine geochemical budgets. *Nature* **458**, 493–496 (2009).

29. M. Dellinger, D. S. Hardisty, N. J. Planavsky, B. C. Gill, B. Kalderon-Asael, D. Asael, T. Croissant, P. K. Swart, A. J. West, The effects of diagenesis on lithium isotope ratios of shallow marine carbonates. *Am. J. Sci.* **320**, 150–184 (2020).

30. J. Roberts, K. Kaczmarek, G. Langer, L. C. Skinner, J. Bijma, H. Bradbury, A. V. Turchyn, F. Lamy, S. Misra, Lithium isotopic composition of benthic foraminifera: A new proxy for paleo-pH reconstruction. *Geochim. Cosmochim. Acta* **236**, 336–350 (2018).

31. N. Vigier, C. Rollion-Bard, Y. Levenson, J. Erez, Lithium isotopes in foraminifera shells as a novel proxy for the ocean dissolved inorganic carbon (DIC). *Compt. Rendus Geosci.* **347**, 43–51 (2015).

32. C. C. Day, P. A. E. Pogge von Strandmann, A. J. Mason, Lithium isotopes and partition coefficients in inorganic carbonates: Proxy calibration for weathering reconstruction. *Geochim. Cosmochim. Acta* **305**, 243–262 (2021).

33. S. Misra, P. N. Froelich, Measurement of lithium isotope ratios by quadrupole-ICP-MS: Application to seawater and natural carbonates. *J. Anal. At. Spectrom.* **24**, 1524–1533 (2009).

34. P. A. E. Pogge von Strandmann, A. Desrochers, M. J. Murphy, A. J. Finlay, D. Selby, T. M. Lenton, Global climate stabilisation by chemical weathering during the Hirnantian glaciation. *Geochem. Perspect. Lett.* **3**, 230–237 (2017).

35. L. Bastian, M. Revel, G. Bayon, A. Dufour, N. Vigier, Abrupt response of chemical weathering to Late Quaternary hydroclimate changes in northeast Africa. *Sci. Rep.* **7**, 44231 (2017).

36. G. Li, A. J. West, Evolution of Cenozoic seawater lithium isotopes: Coupling of global denudation regime and shifting seawater sinks. *Earth Planet. Sci. Lett.* **401**, 284–293 (2014).

37. R. S. Hindshaw, R. Tosca, T. L. Goût, I. Farnan, N. J. Tosca, E. T. Tipper, Experimental constraints on Li isotope fractionation during clay formation. *Geochim. Cosmochim. Acta* **250**, 219–237 (2019).

38. S. Li, R. M. Gaschnig, R. L. Rudnick, Insights into chemical weathering of the upper continental crust from the geochemistry of ancient glacial diamictites. *Geochim. Cosmochim. Acta* **176**, 96–117 (2016).

39. E. W. Stokke, M. T. Jones, J. E. Tierney, H. H. Svensen, J. H. Whiteside, Temperature changes across the Paleocene-Eocene Thermal Maximum—A new high-resolution TEX86 temperature record from the Eastern North Sea Basin. *Earth Planet. Sci. Lett.* **544**, 116388 (2020).

40. R. Wieczorek, M. S. Fantle, L. R. Kump, G. Ravizza, Geochemical evidence for volcanic activity prior to and enhanced terrestrial weathering during the Paleocene Eocene Thermal Maximum. *Geochim. Cosmochim. Acta* **119**, 391–410 (2013).

41. C. J. Hollis, T. D. Jones, E. Anagnostou, P. K. Bijl, M. J. Cramwinckel, Y. Cui, G. R. Dickens, K. M. Edgar, Y. Eley, D. Evans, G. L. Foster, J. Frieling, G. N. Inglis, E. M. Kennedy, R. Kozdon, V. Lauretano, C. H. Lear, K. Littler, L. Lourens, A. N. Meckler, B. D. A. Naafs, H. Pälike, R. D. Pancost, P. N. Pearson, U. Röhl, D. L. Royer, U. Salzmann, B. A. Schubert, H. Seebeck, A. Sluijs, R. P. Speijer, P. Stassen, J. Tierney, A. Tripati, B. Wade, T. Westerhold, C. Witkowski, J. C. Zachos, Y. G. Zhang, M. Huber, D. J. Lunt, The DeepMIP contribution to PMIP4: Methodologies for selection, compilation and analysis of latest Paleocene and early Eocene climate proxy data, incorporating version 0.1 of the DeepMIP database. *Geosci. Model Dev.* **12**, 3149–3206 (2019).

42. P. A. E. Pogge von Strandmann, H. C. Jenkyns, R. G. Woodfine, Lithium isotope evidence for enhanced weathering during Oceanic Anoxic Event 2. *Nat. Geosci.* **6**, 668–672 (2013).

43. J. Pizzuto, J. Keeler, K. Skalak, D. Karwan, Storage filters upland suspended sediment signals delivered from watersheds. *Geology* **45**, 151–154 (2017).

44. A. Tripati, H. Elderfield, Deep-sea temperature and circulation changes at the Paleocene-Eocene Thermal Maximum. *Science* **308**, 1894–1898 (2005).

45. M. Lechler, P. A. E. Pogge von Strandmann, H. C. Jenkyns, G. Prosser, M. Parente, Lithium-isotope evidence for enhanced silicate weathering during OAE 1a (Early Aptian Selli event). *Earth Planet. Sci. Lett.* **432**, 210–222 (2015).

46. N. Coltice, M. Seton, T. Rolf, R. D. Mueller, P. J. Tackley, Convergence of tectonic reconstructions and mantle convection models for significant fluctuations in seafloor spreading. *Earth Planet. Sci. Lett.* **383**, 92–100 (2013).

47. G. Li, H. Elderfield, Evolution of carbon cycle over the past 100 million years. *Geochim. Cosmochim. Acta* **103**, 11–25 (2013).

48. A. J. Dickson, A. S. Cohen, A. L. Coe, M. Davies, E. A. Shcherbinina, Y. O. Gavrilov, Evidence for weathering and volcanism during the PETM from Arctic Ocean and Peri-Tethys osmium isotope records. *Palaeogeogr. Palaeoclimatol. Palaeoecol.* **438**, 300–307 (2015).

49. R. E. Zeebe, LOSCAR: Long-term Ocean-atmosphere-Sediment CArbon cycle Reservoir Model v2.0.4. *Geosci. Model Dev.* **5**, 149–166 (2012).

50. J. Uchikawa, R. E. Zeebe, Influence of terrestrial weathering on ocean acidification and the next glacial inception. *Geophys. Res. Lett.* **35**, L23608 (2008).

51. G. Fontorbe, P. J. Frings, C. L. De la Rocha, K. R. Hendry, D. J. Conley, Constraints on earth system functioning at the paleocene-eocene thermal maximum from the marine silicon cycle. *Paleoceanogr. Paleoclimatol.* **35**, e2020PA003873 (2020).

52. S. R. Gislason, E. H. Oelkers, E. S. Eiriksdottir, M. I. Kardjilov, G. Gisladottir, B. Sigfusson, A. Snorrason, S. Elefsen, J. Hardardottir, P. Torssander, N. Oskarsson, Direct evidence of the feedback between climate and weathering. *Earth Planet. Sci. Lett.* **277**, 213–222 (2009).

53. D. E. Penman, S. K. Turner, P. F. Sexton, R. D. Norris, A. J. Dickson, S. Boulila, A. Ridgwell, R. E. Zeebe, J. C. Zachos, A. Cameron, T. Westerhold, U. Röhl, An abyssal carbonate compensation depth overshoot in the aftermath of the Palaeocene–Eocene Thermal Maximum. *Nat. Geosci.* **9**, 575–580 (2016).

54. L. Sauzéat, R. L. Rudnick, C. Chauvel, M. Garcon, M. Tang, New perspectives on the Li isotopic composition of the upper continental crust and its weathering signature. *Earth Planet. Sci. Lett.* **428**, 181–192 (2015).

55. B. Kisakurek, M. Widdowson, R. H. James, Behaviour of Li isotopes during continental weathering: The Bidar laterite profile, India. *Chem. Geol.* **212**, 27–44 (2004).

56. C. Clergue, M. Dellinger, H. L. Buss, J. Gaillardet, M. F. Benedetti, C. Dessert, Influence of atmospheric deposits and secondary minerals on Li isotopes budget in a highly weathered catchment, Guadeloupe (Lesser Antilles). *Chem. Geol.* **414**, 28–41 (2015).

57. J.-W. Zhang, Z. Q. Zhao, Y. N. Yan, L. F. Cui, Q. L. Wang, J. L. Meng, X. D. Li, C. Q. Liu, Lithium and its isotopes behavior during incipient weathering of granite in the eastern Tibetan Plateau, China. *Chem. Geol.* **559**, 119969 (2021).

58. S. Gudbrandsson, D. Wolff-Boenisch, S. R. Gislason, E. H. Oelkers, Experimental determination of plagioclase dissolution rates as a function of its composition and pH at 22°C. *Geochim. Cosmochim. Acta* **139**, 154–172 (2014).

59. K. Maher, C. I. Steefel, A. F. White, D. A. Stonestrom, The role of reaction affinity and secondary minerals in regulating chemical weathering rates at the Santa Cruz Soil Chronosequence, California. *Geochim. Cosmochim. Acta* **73**, 2804–2831 (2009).

60. E. H. Oelkers, J. Declerqc, G. D. Saldi, S. R. Gislason, J. Schott, Olivine dissolution rates: A critical review. *Chem. Geol.* **500**, 1–19 (2018).

61. N. C. M. Marty, F. Claret, A. Lassin, J. Tremosa, P. Blanc, B. Madé, E. Giffaut, B. Cochepin, C. Tournassat, A database of dissolution and precipitation rates for clay-rocks minerals. *Appl. Geochem.* **55**, 108–118 (2015).

62. L. Yang, C. I. Steefel, Kaolinite dissolution and precipitation kinetics at 22°C and pH 4. *Geochim. Cosmochim. Acta* **72**, 99–116 (2008).

63. N. Vdovic, I. Jurina, S. D. Skapin, I. Sondi, The surface properties of clay minerals modified by intensive dry milling—Revisited. *Appl. Clay Sci.* **48**, 575–580 (2010).

64. F. Macht, K. Eusterhues, G. J. Pronk, K. U. Totsche, Specific surface area of clay minerals: Comparison between atomic force microscopy measurements and bulk-gas (N2) and -liquid (EGME) adsorption methods. *Appl. Clay Sci.* **53**, 20–26 (2011).

65. P. Maffre, Y. Godderis, N. Vigier, J.-S. Moquet, S. Carretier, Modelling the riverine δ7Li variability throughout the Amazon Basin. *Chem. Geol.* **532**, 119336 (2020).

66. G. J. Bowen, D. J. Beerling, P. L. Koch, J. C. Zachos, T. Quattlebaum, A humid climate state during the Palaeocene/Eocene thermal maximum. *Nature* **432**, 495–499 (2004).

67. M. J. Carmichael, R. D. Pancost, D. J. Lunt, Changes in the occurrence of extreme precipitation events at the Paleocene–Eocene thermal maximum. *Earth Planet. Sci. Lett.* **501**, 24–36 (2018).

68. P. M. Sadler, D. J. Jerolmack, in *Strata and Time: Probing the Gaps in Our Understanding*, D. G. Smith, R. J. Bailey, P. M. Burgess, A. J. Fraser, Eds. (Geological Society, 2015), vol. 404, pp. 69–99.

69. C. M. John, S. M. Bohaty, J. C. Zachos, A. Sluijs, S. Gibbs, H. Brinkhuis, T. J. Bralower, North American continental margin records of the Paleocene-Eocene thermal maximum: Implications for global carbon and hydrological cycling. *Paleoceanography* **23** (2008).

70. E. M. Fischer, J. Sedlacek, R. Knutti, Models agree on forced response pattern of precipitation and temperature extremes. *Geophys. Res. Lett.* **41**, 8554–8562 (2014).

71. C. Chen, L. Guerit, B. Z. Foreman, H. J. Hassenruck-Gudipati, T. Adatte, L. Honegger, M. Perret, A. Sluijs, S. Castelltort, Estimating regional flood discharge during Palaeocene-Eocene global warming. *Sci. Rep.* **8**, 13391 (2018).

72. V. Pujalte, J. I. Baceta, B. Schmidtz, A massive input of coarse-grained siliciclastics in the Pyrenean Basin during the PETM: The missing ingredient in a coeval abrupt change in hydrological regime. *Clim. Past* **11**, 1653–1672 (2015).

73. M. A. Nearing, V. Jetten, C. Baffaut, O. Cerdan, A. Couturier, M. Hernandez, Y. le Bissonnais, M. H. Nichols, J. P. Nunes, C. S. Renschler, V. Souchère, K. van Oost, Modeling response of soil erosion and runoff to changes in precipitation and cover. *Catena* **61**, 131–154 (2005).

74. F. F. Pruski, M. A. Nearing, Climate-induced changes in erosion during the 21st century for eight U. S. locations. *Water Resour. Res.* **38**, 34-1–34-11 (2002).

75. D. Favis-Mortlock, J. Boardman, Nonlinear responses of soil erosion to climate change: A modelling study on the UK South Downs. *Catena* **25**, 365–387 (1995).

76. A. Winguth, C. Shellito, C. Shields, C. Winguth, Climate response at the paleocene–Eocene thermal maximum to greenhouse gas forcing—A model study with CCSM3. *J. Clim.* **23**, 2562–2584 (2010).

77. J. J. Armitage, A. C. Whittaker, M. Zakari, B. Campforts, Numerical modelling of landscape and sediment flux response to precipitation rate change. *Earth Surf. Dyn.* **6**, 77–99 (2018).

78. S. R. Gíslason, E. Oelkers, A. Snorrason, Role of river-suspended material in the global carbon cycle. *Geology* **34**, 49–52 (2006).

79. Z. Ma, E. Gray, E. Thomas, B. Murphy, J. Zachos, A. Paytan, Carbon sequestration during the Palaeocene–Eocene Thermal Maximum by an efficient biological pump. *Nat. Geosci.* **7**, 382–388 (2014).

80. M. T. Jones, L. M. E. Percival, E. W. Stokke, J. Frieling, T. A. Mather, L. Riber, B. A. Schubert, B. Schultz, C. Tegner, S. Planke, H. H. Svensen, Mercury anomalies across the Palaeocene–Eocene Thermal Maximum. *Clim. Past* **15**, 217–236 (2019).

81. T. J. Bralower, J. C. Zachos, E. Thomas, M. Parrow, C. K. Paull, D. C. Kelly, I. P. Silva, W. V. Sliter, K. C. Lohmann, Late Paleocene to Eocene paleoceanography of the equatorial Pacific Ocean: Stable isotopes recorded at Ocean Drilling Program Site 865, Allison Guyot. *Paleoceanography* **10**, 841–865 (1995).

82. D. C. Kelly, T. J. Bralower, J. C. Zachos, I. P. Silva, E. Thomas, Rapid diversification of planktonic foraminifera in the tropical Pacific (ODP Site 865) during the late Paleocene thermal maximum. *Geology* **24**, 423–426 (1996).

83. A. K. Tripati, H. Elderfield, Abrupt hydrographic changes in the equatorial Pacific and subtropical Atlantic from foraminiferal Mg/Ca indicate greenhouse origin for the thermal maximum at the Paleocene-Eocene Boundary. *Geochem. Geophys. Geosyst.* **5**, Q02006 (2004).

84. S. Bains, R. M. Corfield, R. D. Norris, Mechanisms of climate warming at the end of the Paleocene. *Science* **285**, 724–727 (1999).

85. K. Takeda, K. Kaiho, Faunal turnovers in central Pacific benthic foraminifera during the Paleocene–Eocene thermal maximum. *Palaeogeogr. Palaeoclimatol. Palaeoecol.* **251**, 175–197 (2007).

86. K. Kaiho, K. Takeda, M. R. Petrizzo, J. C. Zachos, Anomalous shifts in tropical Pacific planktonic and benthic foraminiferal test size during the Paleocene-Eocene thermal maximum. *Palaeogeogr. Palaeoclimatol. Palaeoecol.* **237**, 456–464 (2006).

87. T. Westerhold, U. Röhl, I. Raffi, E. Fornaciari, S. Monechi, V. Reale, J. Bowles, H. F. Evans, Astronomical calibration of the Paleocene time. *Palaeogeogr. Palaeoclimatol. Palaeoecol.* **257**, 377–403 (2008).

88. Y. Cui, L. R. Kump, A. Ridgwell, Initial assessment of the carbon emission rate and climatic consequences during the end-Permian mass extinction. *Palaeogeogr. Palaeoclimatol. Palaeoecol.* **389**, 128–136 (2013).

89. M. T. Jones, L. E. Augland, G. E. Shephard, S. D. Burgess, G. T. Eliassen, M. M. Jochmann, B. Friis, D. A. Jerram, S. Planke, H. H. Svensen, Constraining shifts in North Atlantic plate motions during the Palaeocene by U-Pb dating of Svalbard tephra layers. *Sci. Rep.* **7**, 6822 (2017).

90. K. Piepjohn, W. von Gosen, F. Tessensohn, The Eurekan deformation in the Arctic: An outline. *J. Geol. Soc. Lond.* **173**, 1007–1024 (2016).

91. K. Svennevig, P. Guarnieri, L. Stemmerik, Tectonic inversion in the Wandel Sea Basin: A new structural model of Kilen (eastern North Greenland). *Tectonics* **35**, 2896–2917 (2016).

92. J. Nagy, D. Jargvoll, H. Dypvik, M. Jochmann, L. Riber, Environmental changes during the Paleocene-Eocene Thermal Maximum in Spitsbergen as reflected by benthic foraminifera. *Polar Res.* **32**, 19737 (2013).

93. A. J. Charles, D. J. Condon, I. C. Harding, H. Pälike, J. E. A. Marshall, Y. Cui, L. Kump, I. W. Croudace, Constraints on the numerical age of the Palaeocene-Eocene boundary. *Geochem. Geophys. Geosyst.* **12**, Q0AA17 (2011).

94. R. W. O. B. Knox, J. H. A. Bosch, E. S. Rasmussen, C. Heilmann-Clausen, M. Hiss, I. R. De Lugt, J. Kasinski, C. King, A. Köthe, B. Slodkowska, G. Standke, N. Vandenberghe, in *Petroleum*

Geological Atlas of the Southern Permian Basin Area, H. Dornenbaal, A. Stevenson, Eds. (EAGE Publications, 2010), pp. 211–223.

95. R. W. B. Knox, Tectonic controls on sequence development in the Palaeocene and earliest Eocene of southeast England: Implications for North Sea stratigraphy. *Geol. Soc. Spec. Publ.* **103**, 209–230 (1996).

96. O. R. Clausen, O. B. Nielsen, M. Huuse, O. Michelsen, Geological indications for Palaeogene uplift in the eastern North Sea Basin. *Glob. Planet. Chang.* **24**, 175–187 (2000).

97. C. Heilmann-Clausen, O. B. Nielsen, F. Gersner, Lithostratigraphy and depositional environments in the Upper Paleocene and Eocene of Denmark. *Bull. Geol. Soc. Den.* **33**, 287–323 (1985).

98. A. G. Mitlehner, Palaeoenvironments in the North Sea Basin around the Paleocene-Eocene boundary: Evidence from diatoms and other siliceous microfossils. *Geol. Soc. Spec. Publ.* **101**, 255–273 (1996).

99. J. A. Boyden, D. R. Müller, M. Gurnis, T. H. Torsvik, J. A. Clark, M. Turner, H. Ivey-Law, R. J. Watson, J. Cannon, in *Geoinformatics: Cyberinfrastructure for the Solid Earth Sciences,* G. R. Keller, C. Baru, Eds. (Cambridge Univ. Press, 2011), pp. 95–114.

100. M. Gurnis, M. Turner, S. Zahirovic, L. DiCaprio, S. Spasojevic, R. D. Müller, J. Boyden, M. Seton, V. C. Manea, D. J. Bower, Plate Tectonic reconstructions with continuously closing plates. *Comput. Geosci.* **38**, 35–42 (2012).

101. R. D. Müller, M. Seton, S. Zahirovic, S. E. Williams, K. J. Matthews, N. M. Wright, G. E. Shephard, K. T. Maloney, N. Barnett-Moore, M. Hosseinpour, D. J. Bower, J. Cannon, Ocean basin evolution and global-scale plate reorganization events since pangea breakup. *Annu. Rev. Earth Planet. Sci.* **44**, 107–138 (2016).

102. P. Wessel, W. Smith, R. Scharroo, J. Luis, F. Wobbe, Generic mapping tools: Improved version released. *EOS Trans. Am. Geophys. Union* **94**, 409–410 (2013).

103. J. Golonka, Phanerozoic paleoenvironment and paleolithofacies maps: Cenozoic. *Geol./Akad. Gór.-Hut. Im Stanisława Staszica W Krakowie* **35**, 507–587 (2009).

104. C. S. Marriott, G. M. Henderson, N. S. Belshaw, A. W. Tudhope, Temperature dependence of δ^{7}Li, δ^{44}Ca and Li/Ca during growth of calcium carbonate. *Earth Planet. Sci. Lett.* **222**, 615–624 (2004).

105. M. O. Clarkson, C. H. Stirling, H. C. Jenkyns, A. J. Dickson, D. Porcelli, C. M. Moy, P. A. E. Pogge von Strandmann, I. R. Cooke, T. M. Lenton, Uranium isotope evidence for two episodes of deoxygenation during Oceanic Anoxic Event 2. *Proc. Natl. Acad. Sci. U.S.A.* **115**, 2918–2923 (2018).

106. S. R. C. Remmerzwaal, S. Dixon, I. J. Parkinson, D. N. Schmidt, F. M. Monteiro, P. Sexton, M. A. Fehr, C. Peacock, Y. Donnadieu, R. H. James, Investigating ocean deoxygenation during the PETM through the Cr isotopic signature of foraminifera. *Paleoceanogr. Paleoclimatol.* **34**, 917–929 (2019).

107. M. R. Petrizzo, The onset of the Paleocene–Eocene Thermal Maximum (PETM) at Sites 1209 and 1210 (Shatsky Rise, Pacific Ocean) as recorded by planktonic foraminifera. *Mar. Micropaleontol.* **63**, 187–200 (2007).

108. P. Stoffyn-Egli, F. T. Mackenzie, Mass balance of dissolved lithium in the oceans. *Geochim. Cosmochim. Acta* **48**, 859–872 (1984).

109. P. A. E. Pogge von Strandmann, S. Opfergelt, Y.-J. Lai, B. Sigfússon, S. R. Gislason, K. W. Burton, Lithium, magnesium and silicon isotope behaviour accompanying weathering in a basaltic soil and pore water profile in Iceland. *Earth Planet. Sci. Lett.* **339–340**, 11–23 (2012).

110. P. A. E. Pogge von Strandmann, K. W. Burton, S. Opfergelt, E. S. Eiríksdóttir, M. J. Murphy, A. Einarsson, S. R. Gislason, The effect of hydrothermal spring weathering processes and primary productivity on lithium isotopes: Lake Myvatn, Iceland. *Chem. Geol.* **445**, 4–13 (2016).

111. E. Lemarchand, F. Chabaux, N. Vigier, R. Millot, M. C. Pierret, Lithium isotope systematics in a forested granitic catchment (Strengbach, Vosges Mountains, France). *Geochim. Cosmochim. Acta* **74**, 4612–4628 (2010).

112. B. Kisakürek, R. H. James, N. B. W. Harris, Li and δ^{7}Li in Himalayan rivers: Proxies for silicate weathering? *Earth Planet. Sci. Lett.* **237**, 387–401 (2005).

113. R. Millot, N. Vigier, J. Gaillardet, Behaviour of lithium and its isotopes during weathering in the Mackenzie Basin, Canada. *Geochim. Cosmochim. Acta* **74**, 3897–3912 (2010).

114. L.-F. Gou, Z. Jin, P. A. E. Pogge von Strandmann, G. Li, Y. X. Qu, J. Xiao, L. Deng, A. Galy, Li isotopes in the middle Yellow River: Seasonal variability, sources and fractionation. *Geochim. Cosmochim. Acta* **248**, 88–108 (2019).

115. T. Elliott, A. Thomas, A. Jeffcoate, Y. L. Niu, Lithium isotope evidence for subduction-enriched mantle in the source of mid-ocean-ridge basalts. *Nature* **443**, 565–568 (2006).

116. Y. Huh, L. H. Chan, J. M. Edmond, Lithium isotopes as a probe of weathering processes: Orinoco River. *Earth Planet. Sci. Lett.* **194**, 189–199 (2001).

117. P. A. E. Pogge von Strandmann, K. W. Burton, R. H. James, P. van Calsteren, S. R. Gíslason, F. Mokadem, Riverine behaviour of uranium and lithium isotopes in an actively glaciated basaltic terrain. *Earth Planet. Sci. Lett.* **251**, 134–147 (2006).

118. P. A. E. Pogge von Strandmann, G. M. Henderson, The Li isotope response to mountain uplift. *Geology* **43**, 67–70 (2015).

119. N. Vigier, A. Decarreau, R. Millot, J. Carignan, S. Petit, C. France-Lanord, Quantifying Li isotope fractionation during smectite formation and implications for the Li cycle. *Geochim. Cosmochim. Acta* **72**, 780–792 (2008).

120. J. Wimpenny, C. A. Colla, P. Yu, Q. Z. Yin, J. R. Rustad, W. H. Casey, Lithium isotope fractionation during uptake by gibbsite. *Geochim. Cosmochim. Acta* **168**, 133–150 (2015).

121. J. Wimpenny, S. R. Gíslason, R. H. James, A. Gannoun, P. A. E. Pogge von Strandmann, K. W. Burton, The behaviour of Li and Mg isotopes during primary phase dissolution and secondary mineral formation in basalt. *Geochim. Cosmochim. Acta* **74**, 5259–5279 (2010).

122. J. S. Pistiner, G. M. Henderson, Lithium-isotope fractionation during continental weathering processes. *Earth Planet. Sci. Lett.* **214**, 327–339 (2003).

123. L. H. Chan, J. M. Edmond, G. Thompson, K. Gillis, Lithium isotopic composition of submarine basalts: Implications for the lithium cycle in the oceans. *Earth Planet. Sci. Lett.* **108**, 151–160 (1992).

124. L. H. Chan, J. M. Gieskes, C. F. You, J. M. Edmond, Lithium isotope geochemistry of sediments and hydrothermal fluids of the Guaymas Basin, Gulk of California. *Geochim. Cosmochim. Acta* **58**, 4443–4454 (1994).

125. C. V. Ullmann, H. J. Campbell, R. Frei, S. P. Hesselbo, P. A. E. Pogge von Strandmann, C. Korte, Partial diagenetic overprint of Late Jurassic belemnites from New Zealand: Implications for the preservation potential of δ7Li values in calcite fossils. *Geochim. Cosmochim. Acta* **120**, 80–96 (2013).

126. E. E. Angino, G. K. Billings, Lithium content of sea water by atomic absorption spectrometry. *Geochim. Cosmochim. Acta* **30**, 153–158 (1966).

127. J. D. Hem, Study and interpretation of the chemical characteristics of natural waters. *U.S. Geol. Surv. Water Supply Pap.* **2254** (1985).

128. Y. Huh, L. H. Chan, L. Zhang, J. M. Edmond, Lithium and its isotopes in major world rivers: Implications for weathering and the oceanic budget. *Geochim. Cosmochim. Acta* **62**, 2039–2051 (1998).

129. Q.-L. Wang, B. Chetelat, Z. Q. Zhao, H. Ding, S. L. Li, B. L. Wang, J. Li, X. L. Liu, Behavior of lithium isotopes in the Changjiang River system: Sources effects and response to weathering and erosion. *Geochim. Cosmochim. Acta* **151**, 117–132 (2015).

130. N. Vigier, S. R. Gislason, K. W. Burton, R. Millot, F. Mokadem, The relationship between riverine lithium isotope composition and silicate weathering rates in Iceland. *Earth Planet. Sci. Lett.* **287**, 434–441 (2009).

131. S. Henchiri, C. Clergue, M. Dellinger, J. Gaillardet, P. Louvat, J. Bouchez, The influence of hydrothermal activity on the Li isotopic signature of rivers draining volcanic areas. *Proc. Earth Planet. Sci.* **10**, 223–230 (2014).

132. P. A. E. Pogge von Strandmann, K. W. Burton, R. H. James, P. van Calsteren, S. R. Gislason, Assessing the role of climate on uranium and lithium isotope behaviour in rivers draining a basaltic terrain. *Chem. Geol.* **270**, 227–239 (2010).

133. R. A. Berner, GEOCARB II; A revised model of atmospheric CO 2 over Phanerozoic time. *Am. J. Sci.* **294**, 56–91 (1994).

134. G. Colbourn, A. Ridgwell, T. M. Lenton, The Rock Geochemical Model (RokGeM) v0.9. *Geosci. Model Dev.* **6**, 1543–1573 (2013).

135. R. L. Rudnick, P. B. Tomascak, H. B. Njo, L. R. Gardner, Extreme lithium isotopic fractionation during continental weathering revealed in saprolites from South Carolina. *Chem. Geol.* **212**, 45–57 (2004).
